# In Vitro Assessment of the Neuroprotective Effects of Pomegranate (*Punica granatum* L.) Polyphenols Against Tau Phosphorylation, Neuroinflammation, and Oxidative Stress

**DOI:** 10.3390/nu16213667

**Published:** 2024-10-28

**Authors:** Mehdi Alami, Kaoutar Boumezough, Echarki Zerif, Nada Zoubdane, Abdelouahed Khalil, Ton Bunt, Benoit Laurent, Jacek M. Witkowski, Charles Ramassamy, Samira Boulbaroud, Tamas Fulop, Hicham Berrougui

**Affiliations:** 1 Department of Biology, Polydisciplinary Faculty, University Sultan Moulay Slimane, Beni Mellal 23020, Morocco; mehdi.alami@usherbrooke.ca (M.A.); kaoutarrosa1@gmail.com (K.B.); sboulbaroud@gmail.com (S.B.); 2 Research Center on Aging, Faculty of Medicine and Health Sciences, University of Sherbrooke, Sherbrooke, QC J1H 4N4, Canada; echarki.zerif@umontreal.ca (E.Z.); zoubdane.nada@usherbrooke.ca (N.Z.); a.khalil@usherbrooke.ca (A.K.); benoit.laurent@usherbrooke.ca (B.L.); tamas.fulop@usherbrooke.ca (T.F.); 3 Izumi Biosciences, Inc., Lexington, MA 02420, USA; tbunt@izumibiosciences.com; 4 Department of Embryology, Medical University of Gdansk, 80-210 Gdańsk, Poland; jawit@gumed.edu.pl; 5 INRS-Centre Armand-Frappier Santé Biotechnologie, Laval, QC H7V 1B7, Canada; ramassamy@inrs.ca

**Keywords:** neuroinflammation, Alzheimer’s disease, pomegranate (*Punica granatum* L.), oxidative stress, microglia, amyloid-beta, phospho-Tau-181, ellagic acid, punicalagin

## Abstract

Background: Oxidative stress and chronic inflammation, at both the systemic and the central level, are critical early events in atherosclerosis and Alzheimer’s disease (AD). Purpose: To investigate the oxidative stress-, inflammation-, and Tau-phosphorylation-lowering effects of pomegranate polyphenols (PPs) (punicalagin, ellagic acid, peel, and aril extracts). Methods: We used flow cytometry to quantify the protein expression of proinflammatory cytokines (IL-1β) and anti-inflammatory mediators (IL-10) in THP-1 macrophages, as well as M1/M2 cell-specific marker (CD86 and CD163) expression in human microglia HMC3 cells. The IL-10 protein expression was also quantified in U373-MG human astrocytes. The effect of PPs on human amyloid beta 1-42 (Aβ_1-42_)-induced oxidative stress was assessed in the microglia by measuring ROS generation and lipid peroxidation, using 2′,7′-dichlorofluorescein diacetate (DCFH-DA) and thiobarbituric acid reactive substance (TBARS) tests, respectively. Neuronal viability and cell apoptotic response to Aβ_1-42_ toxicity were assayed using the MTT (3-(4,5-dimethylthiazolyl-2)-2,5-diphenyltetrazolium bromide) assay and the annexin-V-FITC apoptosis detection kit, respectively. Finally, flow cytometry analysis was also performed to evaluate the ability of PPs to modulate Aβ_1-42_-induced Tau-181 phosphorylation (pTau-181). Results: Our data indicate that PPs are significantly (*p* < 0.05) effective in countering Aβ_1-42_-induced inflammation through increasing the anti-inflammatory cytokines (IL-10) in U373-MG astrocytes and THP1 macrophages and decreasing proinflammatory marker (IL-1β) expression in THP1 macrophages. The PPs were also significantly (*p* < 0.05) effective in inducing the phenotypic transition of THP-1 macrophages and microglial cells from M1 to M2 by decreasing CD86 and increasing CD163 surface receptor expression. Moreover, our treatments have a significant (*p* < 0.05) beneficial impact on oxidative stress, illustrated in the reduction in TBARS and ROS generation. Our treatments have significant (*p* < 0.05) cell viability improvement capacities and anti-apoptotic effects on human H4 neurons. Furthermore, our results suggest that Aβ_1-42_ significantly (*p* < 0.05) increases pTau-181. This effect is significantly (*p* < 0.05) attenuated by arils, peels, and punicalagin and drastically reduced by the ellagic acid treatment. Conclusion: Overall, our results attribute to PPs anti-inflammatory, antioxidant, anti-apoptotic, and anti-Tau-pathology potential. Future studies should aim to extend our knowledge of the potential role of PPs in Aβ_1-42_-induced neurodegeneration, particularly concerning its association with the tauopathy involved in AD.

## 1. Introduction

It was necessary to wait until 1988 so that Rogers and coworkers could highlight the involvement of the immune response in the etiology of Alzheimer’s disease (AD) [1]. Neuroinflammation and the neuroimmune response in AD involve astrocytes and microglia activation. Astrocytes are multifunctional “housekeeping” cells and the most abundant type of glial cells in the central nervous system (CNS). These dynamic regulators ensure vital functions such as brain water homeostasis [2], blood flow regulation [3], dynamic synaptic structure [4], extracellular pH regulation [5], and brain-free radical species [6] detoxification. Microglia cells are dynamic immunocompetent cells and “brain-resident macrophages” that phagocyte neuronal debris and clean aggregated amyloid-beta (Aβ) fragments [7]. They maintain brain homeostasis by ensuring critical CNS activities, including brain development [8], neuronal survival [9], neurogenesis, and synaptic remodelling [10].

In injured or diseased conditions, these cells become chronically activated and adopt the M1 phenotype to express several cell surface receptors, such as CD80 and CD86, as well as a variety of reactive oxygen species, proinflammatory cytokines (interleukin-1 beta (IL-1β), interleukin-6 (IL-6), interferon-alpha (INF-α), and tumor necrosis factor-alpha (TNF-α)), and chemokines.

In most cases, microglial cells can accelerate the healing process and strengthen recovery. However, for various reasons, including chronic exposure to Aβ [11] due to its overexpression or due to the failure of its clearance by microglia cells, the M1 phenotype may persist, leading to brain injury, chronic neuroinflammation, oxidative stress, reduced neuronal viability, and brain atrophy. Mechanistically, soluble Aβ can bind numerous microglia molecular-pattern recognition and G protein-coupled receptors, such as toll-like and formyl peptide receptors, to directly induce MAPK/Erk and NFκB signaling pathways [12,13]. This leads to the expression of many proinflammatory genes, such as TNFα, IL-1β, and IL6 [14]. 

Consequently, Aβ-peptide targets neuroinflammation [15], oxidative stress [16], blood–brain barrier transporter disruption [17], neuronal apoptosis [18], and Tau phosphorylation [19], as well as enhancing astrocyte reactivity [20]. All these events appear to be causative alterations in AD. On the other hand, the implication of neuroinflammation in AD progression is also supported by numerous epidemiological studies, which indicate that the intake of non-steroidal anti-inflammatory drugs (NSAIDs) can reduce the risk of developing AD [21,22]. In addition, anti-inflammatory therapies were reported to be effective in altering Aβ processing and deposition [23], providing additional proof and pointing to the aberrant involvement of neuroinflammation in AD etiology.

Systemic inflammation is an immune process that localizes, opsonizes, and eliminates toxic compounds, pathological aggressors, and the source of insult. Failure of this healing process results in chronic inflammation, which, over time, may lead to numerous chronic inflammatory diseases, such as cancer, neurodegenerative diseases, and atherosclerosis. Atherosclerosis is a multi-stage process of arterial plaque formation and an immunoinflammatory disease that affects large and medium-sized arteries, causing fatal complications such as heart attack, vascular dementia, and stroke. Macrophages’ plasticity and their M1/M2 phenotypes contribute to atherosclerosis. The M1 macrophage phenotype seems to be adopted in response to exogenous/endogenous stressors, while the M2 phenotype is generated to help tissue repair, modulate inflammatory phases, and accelerate the recovery process. 

It has been shown that peripheral inflammation can induce neuroinflammation [24,25], which in turn can lead to severe mental illness and neurodevelopmental problems [26]. In fact, systemic proinflammatory mediators can modulate the neuronal microenvironment, leading to the activation of microglial cells [24,25]. Recent data suggest that persistent inflammation can induce leakage and dysfunction in the blood–brain barrier (BBB) [27], significantly reducing BBB selectivity. This decline results in the organism’s inability to adequately manage its inflammatory state, leading to various pathological consequences, such as reduced brain–blood barrier selectivity [28] and the death of glial and neuronal cells [29,30]. This gradual deterioration of brain homeostasis can result in progressive cognitive and behavioral deficits and point to a strong link between peripheral and central inflammation.

To partially recapitulate the mentioned M1 pathological aspects, we exposed HMC3 human microglial, U373-MG astrocytes, and macrophages derived from human THP1 monocytes, to a variety of stimuli, including Aβ_1-42_ and LPS. These cell lines were employed in this study due to their immune functions, which respond to our study question, as they were previously used to create polarized cellular models for the M1 phenotype [31,32,33]. H4 cells are a neuronal cell line previously confirmed to produce phospho-Tau protein [34].

Polyphenols and phytochemicals play a role in plants’ immune systems, providing toxicity-based protection against external aggressors, including bacteria, fungi, and insects. However, these active molecules do not mediate harmful effects on human health, as the ingested amount is considered as subtoxic. In addition, at low concentrations, these dietary antioxidants were reported to exert neuroprotection through the activation of vitagenes and the modulation of hormetic pathways [35,36,37,38].

Ellagitannin-rich foods and diets rich in polyphenols have received considerable attention due to their correlation with a lower risk of developing several chronic inflammatory and oxidative-stress-related diseases [39,40,41]. As our team found previously [33], pomegranate (*Sefri* variety) contains various active phytochemicals, such as anthocyanins, flavonoids, gallic acid, ellagic acid, α and β-punicalagin. In addition, we demonstrated that pomegranate peels and arils phenolic extracts can inhibit reactive oxygen species (ROS) and conjugated diene formation and improve paraoxonase 1 (PON1) expression and activity [42]. However, their neuroprotective, central anti-inflammatory impact and possible preventive effects against AD-related neuroinflammation have not been sufficiently studied. To the best of our knowledge, only a few published papers [43,44,45,46,47,48] have focused their research interest on investigating PP effects on neuroinflammation, and only one study has evaluated the impact of pomegranate urolithin, an ellagic acid derivative, on p-Tau [49]. These studies did not fully cover the subject, and many critical aspects of this topic still need to be investigated. These include the effects of punicalagin and ellagic acid, a potent pomegranate-derived active molecule with remarkable antioxidant and anti-inflammatory properties, on Aβ-induced Tau phosphorylation, as well as their impact on the phenotypic transition of microglia from M1 to M2-state. Therefore, a considerable need exists to cover this gap and explore pomegranate’s bioeffects on molecular and physiological processes leading to CNS degeneration.

## 2. Materials and Methods

### 2.1. Chemicals and Reagents

Tert butyl hydroperoxide (TBHP), 2-thiobarbituric acid (TBA), hydrochloric acid (HCL), butylated hydroxytoluene (BHT), radioimmunoprecipitation assay buffer (RIPA), dimethyl sulfoxide (DMSO), and 3-(4,5-dimethylthiazolyl-2)-2,5-diphenyltetrazolium bromide (MTT) were purchased from Sigma-Aldrich (St. Louis, MO, USA), except for lipopolysaccharide (LPS) and elacridar, which were from Sigma-Aldrich Ltd. (Oakville, ON, Canada). Trichloroacetic acid (TCA) and butanol were from Fisher Scientific (Loughborough, UK). Punicalagin and ellagic acid were purchased from Cayman Chemical (Ann Arbor, MI, USA). Human beta-amyloid-(1-42) was supplied by Abcam (Toronto, ON, Canada). Dichlorodihydrofluorescein diacetate (H2-DCFH-DA) was purchased from Invitrogen (Waltham, MA, USA). Annexin V-FITC apoptosis detection assay kit was purchased from BioLegend. Pierce BCA protein assay kit was from Thermo Fisher Scientific (Waltham, MA, USA). Ethanol, phorbol myristate acetate (PMA), and bovine serum albumin were from Wisent Inc. (Saint-Jean-Baptiste, QC, Canada). 

### 2.2. Plant Material

Moroccan pomegranate (*Punica granatum* L.) fruits of the *Sefri* variety were grown and harvested at a local farm in the Beni Mellal-Khenifra region, Oulad Abdallah area (Central Morocco; Latitude: 23°50′05″ E; Longitude: 6°48′98″ N). The authenticity of our variety was confirmed by Professor Abbas Younes, taxonomist and professor at Sultan Moulay Slimane University.

#### Pomegranate Polyphenol Extraction and Chemical Characterization

Pomegranate peel and arils are rich in phenolic compounds, and our team has previously attributed therapeutic activities to these phytochemicals [42]. Peel and aril polyphenol extracts were prepared as previously described by Benchagra et al. [42]. Briefly, aril molasses and peel powder were subjected to extraction (methanol/water, 70:30) for 48 h at 4 °C. The hydroalcoholic extracts were centrifuged, filtered, lyophilized, and stored at −20 °C until use. We have previously determined the chemical content of total flavonoids, total anthocyanins, and total polyphenols and specific polyphenols by HPLC analysis of aril and peel phenolic extracts [42].

### 2.3. Human Aβ_1-42_ Preparation

Human Aβ_1-42_ peptide was supplied by Abcam company (MW 4514.08; AB120301), and it was dissolved in DMSO (0.1%) and diluted in PBS to a final concentration of 1 mg/mL. Next, it was immediately sonicated for 15 min at room temperature and centrifuged at 15,000× *g* at 4 °C for 20 min. The supernatant was stored at −80 °C in 50 µL aliquots until use, as previously described [50].

### 2.4. Cell Culture

Human H4 neuroglioma (ATCC HTB-148), HMC3 immortalized human microglial cell line (ATCC CRL-3304), and human THP-1 monocytes (ATCC TIB-202) were purchased from the American Type Culture Collection (Manassas, VA, USA) via Cederlane^®^ company (Burlington, ON, Canada). THP1 cells were differentiated into macrophages using PMA. H4, HMC3, and THP-1 cell lines were cultured in Dulbecco’s modified Eagle’s medium (DMEM), Eagle’s Minimum Essential Medium (EMEM), and Roswell Park Memorial Institute (RPMI) medium, respectively. The U373-MG human astrocyte cell line was kindly provided by Professor Denis at the University of Sherbrooke, Canada, and it was grown in DMEM. All mediums were supplemented with 10% (*v*/*v*) heat-inactivated fetal bovine serum, 10 U/mL penicillin, and 10 μg/mL of streptomycin. The used cells were in passages between 3 and 6, and were maintained in a 5% CO_2_ humidified incubator at 37 °C.

### 2.5. Cell Viability Measurement

The effects of human Aβ_1-42_ and PP on cell growth were studied using MTT test. The MTT assay is a colorimetric measurement that uses metabolic activity as an indicator of cell viability. Briefly, H4 neurons were seeded in 96-well plates (10^4^ cells/well) and incubated for 24 h with 20 µg/mL of Aβ_1-42_, either in the presence of PPs or not. The medium was removed, and cells were incubated for 2 h with MTT (0.5 mg/mL). After washing, DMSO-dissolved formazan crystals formed, and the absorbance was recorded at 570 nm using a microplate reader (PerkinElmer, Woodbridge, ON, Canada) [51]. 

### 2.6. Cell Apoptosis Assay

Apoptotic cells were detected using the FITC annexin-V apoptosis detection kit according to the manufacturer’s instructions. In brief, H4 neurons were incubated with or without 15 µg/mL of Aβ_1-42_ in the presence or absence of PP for 24 h. All cells were collected, washed with PBS, and stained with 5 µL of FITC-conjugated annexin-V (20 °C, in the dark for 15 min). Next, cells received 400 µL of annexin-V binding buffer. Data were acquired using flow cytometry (Beckman Coulter, Brea, CA, USA) and analyzed by FlowJo 10.2 software (Tree Star Inc., Ashland, OR, USA).

### 2.7. Measurement of Intracellular ROS

Oxidative stress imbalance is one of the leading causes of various disorders and illnesses, and it is well documented that it is implicated in the pathogenesis of several neurodegenerative diseases, including AD [52]. This assay measured intracellular ROS using the fluorescent probe dichlorodihydrofluorescein diacetate (DCFH-DA), as previously described [53]. Briefly, 2 × 10^5^ HMC3 microglial cells were seeded in a 24-well plate in a complete EMEM medium for 24 h to stabilize at 37 °C. The cells were then pre-treated with PP for 2 h prior to their treatment with human Aβ_1-42_ for an additional 4 h. The cells were washed and incubated with 10 µM of DCFH-DA solution at 37 °C in dark for 30 min. After removing DCFH-DA and washing, protein analysis was conducted using BCA assay according to the manufacturer’s recommendations (Thermo Fisher Scientific). The DFC fluorescence intensity of the cells was measured at excitation and emission wavelengths of 485 nm and 530 nm, respectively, using a VICTOR Multilabel Plate Reader (PerkinElmer, Canada). Results were expressed as fluorescence emission intensity/mg of protein for each condition.

### 2.8. Lipid Peroxidation Assay

Lipids are the major biological components targeted by ROS, leading to cellular damage and injury. Increasing proof suggests that lipid peroxidation is among the early oxidative modifications observed in AD [54]. In this assay, we tested the ability of our treatments to prevent lipid peroxidation. Briefly, HMC3 cells were seeded in 24-well plates and were subjected to PP treatments at different concentrations for 24 h before incubation with TBHP (200 µM) for 1 h. Cell-free supernatants were added to 300 µL of a mixture of (0.5% TBA, 30% TCA, 0.33 M HCL, and 0.005% of butylated hydroxytoluene) and boiled for 60 min. The samples were cooled and 300 µL of the butanol was added to each vial for MDA-TBA extraction. After centrifugation, 100 µL of the butanol fraction was transferred to 96-well plates designed for fluorescence-based assays (PerkinElmer, Canada) to quantify the fluorescence intensity at excitation and emission wavelengths of 530 and 590 nm, respectively [55]. The obtained data were normalized using BCA assay. 

### 2.9. M1/M2 Polarization of Microglia Cells and Systemic Macrophages

Macrophage-mediated inflammation, at both a systemic and a central level, was suggested as a potential contributor to the etiology of AD [56,57]. We attempted to evaluate the ability of our treatments to push M1/M2 shifting in HMC3 and THP1 cells. We investigated the expression of CD86 and CD163 cell surface receptors as M1- and M2-specific markers, respectively. Briefly, cells were stimulated by LPS (1 µg/mL) and cotreated simultaneously with pomegranate extracts (peels or arils) or with pomegranate-purified compounds (punicalagin or ellagic acid) at increasing concentrations for 24 h. Next, cells were harvested and washed twice with cold PBS (1×) and centrifuged (350× *g*, at 4 °C, for 6 min). Subsequently, they were stained for CD86 and CD163 cell surface receptor expression, using FITC-mouse anti-human CD86 and BV711-mouse anti-human CD163 monoclonal antibodies (BD Biosciences, Franklin Lakes, NJ, USA), for 50 min, at 4 °C, in darkness. Following this period, the samples were rewashed, centrifuged, and resuspended in cold PBS for cytometry analysis. Data were collected using a CytoFLEX instrument (Beckman Coulter, Brea, CA, USA) and analyzed by FlowJo 10.2 software (Tree Star Inc., Ashland, OR, USA).

### 2.10. The Assessment of Interleukin 1-Beta (IL-1β) and Interleukin-10 (IL-10) Protein Expression

The U373-MG human astrocytes and THP-1 macrophages were subjected to intracellular staining to quantify the protein expression of IL-10 and IL-1β, according to the manufacturer’s instructions (BD Biosciences, Canada). Briefly, cells were stimulated by LPS (1 µg/mL) and cotreated simultaneously with increasing concentrations of PP. Cells were treated during the last 4 h of culture with protein transport inhibitor A (Brefeldin A: (1:1000); Abcam, Canada) to allow the intracytoplasmic accumulation of cytokines. Next, cells were fixed/permeabilized (1 h at 4 °C, in darkness) using Fix/perm buffer (BD Biosciences). Cells were stained with monoclonal APC-rat anti-human IL-10 (BD Biosciences) and PE-mouse anti-human IL-1β (BD Biosciences) for 50 min at 4 °C, in darkness. Data were collected using a CytoFLEX instrument (Beckman Coulter, Brea, CA, USA) and analyzed by FlowJo 10.2 software (Tree Star Inc., Ashland, OR, USA).

### 2.11. Tau Phosphorylation at Threonine 181

Tau protein is one of the most heavily studied proteins in AD, and the accumulated data point to a considerable contribution of its modified forms (especially by hyperphosphorylation) in AD progression [58]. Human H4 neuroglioma cells were cultured in a 6-well plate at a concentration of 1 × 10^6^ and grew to about 80% of confluence. All cells, except the control group, were stimulated with 10 µg/mL of human Aβ_1-42_ and simultaneously cotreated, or not, with PPs for 24 h. Subsequently, cells were fixed/permeabilized (1 h at 4 °C, in darkness) using Fix/perm buffer (BD Biosciences) and stained with primary rabbit monoclonal anti-phospho-Tau181 antibody (Abcam Canada) for an additional 1 h. After washing, the unconjugated anti-phospho-Tau181 antibody was conjugated with Alexa-Fluor 647 anti-rabbit secondary antibody (Invitrogen) for 45 min at 4 °C, in darkness. Following this period, the samples were washed twice, centrifuged, and resuspended in cold-PBS for cytometry analysis. Data were collected using a CytoFLEX instrument (Beckman Coulter, Brea, CA, USA) and analyzed by FlowJo 10.2 software (Tree Star Inc., Ashland, OR, USA).

### 2.12. Statistical Analysis

The statistical analysis was performed using version 10.2.3 of GraphPad Prism software (GraphPad Software^®^, Inc., La Jolla, CA, USA), and the obtained results were expressed as mean ± SEM. Differences between groups were analyzed using a *t*-test (two groups) or one-way ANOVA with Dunnett’s multiple comparisons test. The significance levels were * *p* < 0.05, ** *p* < 0.01, *** *p* < 0.001.

## 3. Results and Discussion

### 3.1. Phytochemical Analysis

Our previously published results [42] demonstrated that peel extracts are richer than aril extracts in total phenolic and flavonoid compounds, and no significant difference was observed in anthocyanins content. Moreover, the HPLC analysis showed that both extracts are especially rich in α-punicalagin and β-punicalagin, but also contain ellagic and gallic acids, with a higher proportion in peel than in aril extracts [42].

### 3.2. Cell Viability Measurement

**Figure 1:**
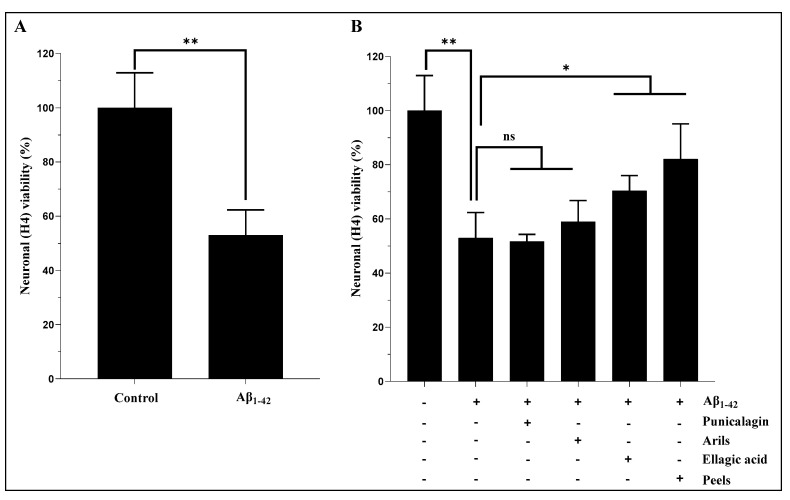
The modulatory effects of peels, arils, punicalagin, and ellagic acid on human Aβ_1-42_-induced neuronal cytotoxicity. Neuronal H4 cells were exposed, or not, to 20 µg/mL of Aβ_1-42_ in the presence or absence of the above treatments for 24 h. (**A**) represents the effect of Aβ_1-42_ on the neuronal cells viability. (**B**) illustrates the effects of PPs (peels: 200 µg/mL; arils: 200 µg/mL; punicalagin: 50 µg/mL; and ellagic acid: 50 µM) on the Aβ_1-42_-related cytotoxicity. Data are expressed as means ± SEM. (*) vs. Aβ_1-42_: * *p* < 0.05, ** *p* < 0.01.

It is believed that increased concentration of Aβ is one of the leading causes of synaptic degeneration, exposing the brain to irreversible neuronal loss and regional atrophy [59]. We first investigated the cytotoxic effect of human Aβ_1-42_ by measuring the metabolic activity of H4 neurons and, subsequently, the cytoprotective effects of PPs. The used concentration was fixed based upon results obtained from a dose-dependent experiment that we carried out with increased concentrations of Aβ_1-42_ (5 to 20 µg/mL, Figure S1). The obtained data show that the Aβ_1-42_ treatments resulted in a significant (*p* < 0.0001) decrease in cell viability in all the used concentrations (5 to 20 µg/mL), with significant cytotoxicity at 20 µg/mL. Therefore, we tested the ability of our treatments to improve neuronal viability in the presence of this concentration (20 µg/mL). Our results in Figure 1A show that human Aβ_1-42_ induce a significant decrease in neuronal viability (*p* < 0.01). However, the treatment with PPs significantly attenuates this cytotoxic effect (Figure 1B). As illustrated in Figure 1B, ellagic acid and pomegranate peel polyphenols, but not punicalagin and pomegranate aril treatments, attenuated the cytotoxic effects of human Aβ_1-42_ on H4 neurons. The peel treatment appears to be more potent in protecting cells against Aβ_1-42_. On the other hand, no cytotoxic effect was observed under the PP treatments when used alone (Figure S2). Previously published reports agree with these findings on PPs’ ability to improve cell viability. The data from the study by Dasilva et al. [45] showed that pomegranate urolithin, a derivative compound of ellagic acid, can improve SH-SY5Y viability and attenuate LPS-induced toxicity. In addition, pomegranate peel extract increased cell viability and showed neuroprotective effects against cisplatin-induced neurotoxicity in a dose-dependent manner [44,60]. Moreover, Fathy and coworkers [61] demonstrated that pomegranate arils and juice reduced paraquat-induced cellular neurotoxicity in a mouse model of Parkinson’s disease. Furthermore, punicalagin was shown to suppress LPS-mediated cytotoxicity in BV2 microglial cells [60]. All these results confirm the safety of PPs and their ability to improve cell survival.

**Figure 2:**
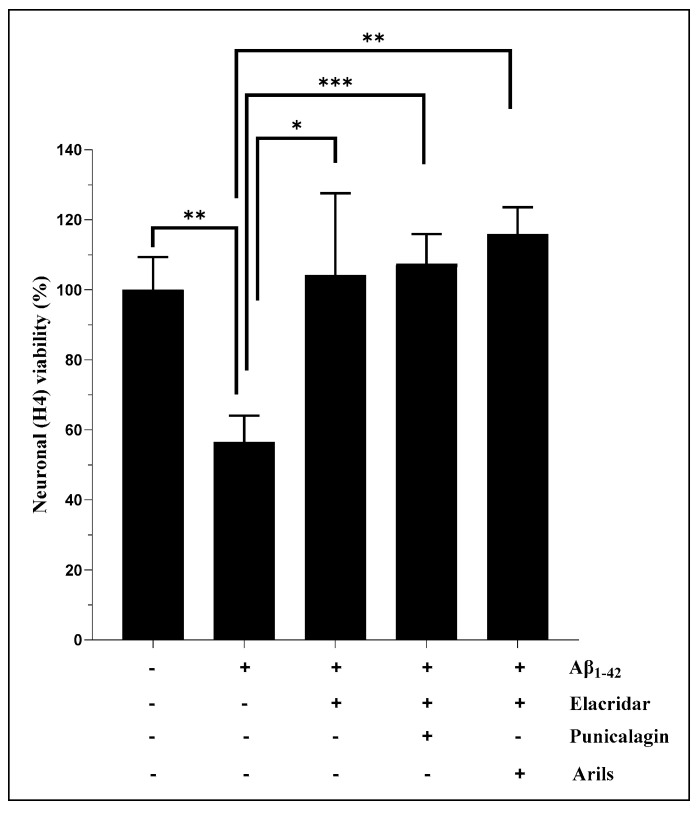
Elacridar improves the effect of pomegranate-arils-rich phenolic extract and punicalagin polyphenols against human-Aβ_1-42_-induced neuronal death. H4 cells were stimulated, or not, with Aβ_1-42_ (20 µg/mL) for 24 h, and simultaneously treated with pomegranate arils extract (200 µg/mL) or punicalagin (50 µg/mL) in the presence of elacridar (500 ng/mL). Data are expressed as means ± SEM. (*) vs. Aβ_1-42_: * *p* < 0.05, ** *p* < 0.01, *** *p* < 0.001.

The aril extract and punicalagin treatments, which had no significant effect (*p* > 0.05) on Aβ_1-42_-mediated neuronal death (Figure 1B), were combined with elacridar, a third-generation P-gp inhibitor and potent bioenhancer that targets multiple drug resistance, to potentially improve outcomes. Indeed, this intervention significantly (*p* < 0.01) improved neuronal viability (Figure 2), which was reflected by the enhancement of cellular respiration and metabolic activity. In fact, the arils + elacridar treatment significantly attenuated the cytotoxic effect of Aβ_1-42_, suggesting a powerful synergistic action between pomegranate aril extract polyphenols and elacridar. Similarly, combining punicalagin with elacridar significantly enhanced the protective effect of punicalagin against Aβ_1-42_-induced neuronal death (Figure 2). Therefore, a co-therapy using natural molecules and elacridar could be envisaged to potentiate their biological effects.

To confirm our findings regarding the viability-improvement effect of our treatments, and to validate the neurotoxic impact of Aβ_1-42_ against H4 neurons, we performed an annexin-V assay to measure neuronal apoptosis.

### 3.3. Human Aβ_1-42_ Induces Apoptosis of H4 Neurons

**Figure 3:**
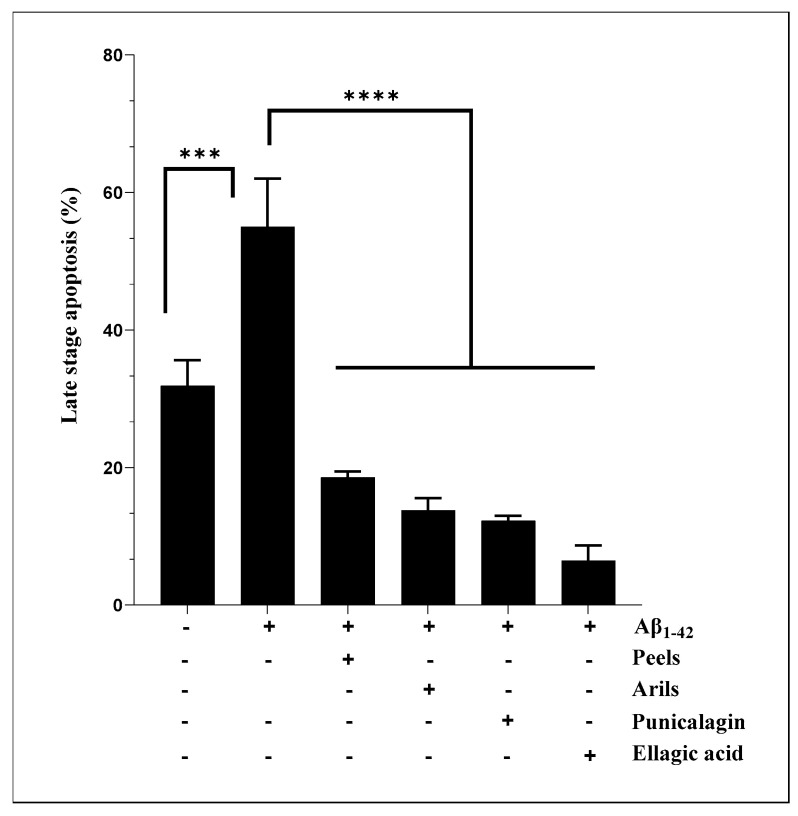
The neuronal-survival-enhancement effect of pomegranate peels (200 µg/mL), arils (200 µg/mL), punicalagin (50 µg/mL), and ellagic acid (50 µM). The H4 neurons were incubated with or without 15 µg/mL of human Aβ_1-42_ for 24 h, in the presence or absence of the above treatments. The obtained results are expressed as mean ± SEM. (*) vs. Aβ_1-42_: *** *p* < 0.001; **** *p* < 0.0001.

Cell apoptosis is an evolutionarily conserved process and a cellular response to injurious stimuli. Previously, a molecular analysis showed that the dying neurons in AD exhibit specific features of apoptosis, including DNA fragmentation [62]. It has been demonstrated that Aβ targets apoptotic pathways, accelerating neuronal loss [63]. Our results indicate that Aβ_1-42_ significantly (*p* < 0.05) increases neuronal apoptosis. These results align with those published by Brouillette et al. [64] and Cizas et al. [65] who found that repeated neuronal exposition to Aβ_1-42_ induces neuronal loss. Additionally, Aβ_1-42_ has been suggested to trigger neurodegeneration via age-dependent autophagic–lysosomal damage [66] and was correlated with brain atrophy in many scientific reports [67,68,69]. This neuronal toxicity was significantly (*p* < 0.001) attenuated by PPs (peels, arils, punicalagin, and ellagic acid) (Figure 3), suggesting an anti-apoptotic effect of PPs against cellular degeneration caused by Aβ_1-42_. The ellagic acid was more effective in reducing apoptosis than the peels and punicalagin, while no significant difference was observed between the ellagic acid and the arils (*p* > 0.05). The peels and punicalagin showed comparable effects (*p* > 0.05). Interestingly, this confirms and supports the reported MTT results in Figure 1. From a mechanistic perspective, ellagic acid can trigger and alter intrinsic apoptotic pathways by inhibiting caspase-3 and caspase-9 [45,70], serine–aspartyl proteases essential in triggering programmed cellular death, to increase neuronal viability. Similarly, it has been shown [60] that pomegranate-peel-phenolic-rich extracts reduce caspase-3 and caspase-9 mRNA transcription, and other potent pomegranate flavonoids, such as quercetin, and pomegranate non-flavonoids, including resveratrol, also exert neuroprotective effects against neuronal apoptosis [71]. Moreover, recent in vivo evidence suggests that pomegranate arils reduce paraquat-enhanced apoptosis [61]. The anti-apoptotic effect of punicalagin was reported by El-Missiry et al. [72], who indicated that punicalagin reduces Bax/Bcl2 and caspase-3 and caspase-9. Based on these findings, pomegranate consumption can be suggested as an effective strategy and a complementary medicine that could limit neuronal degeneration.

### 3.4. Measurement of Intracellular ROS

**Figure 4:**
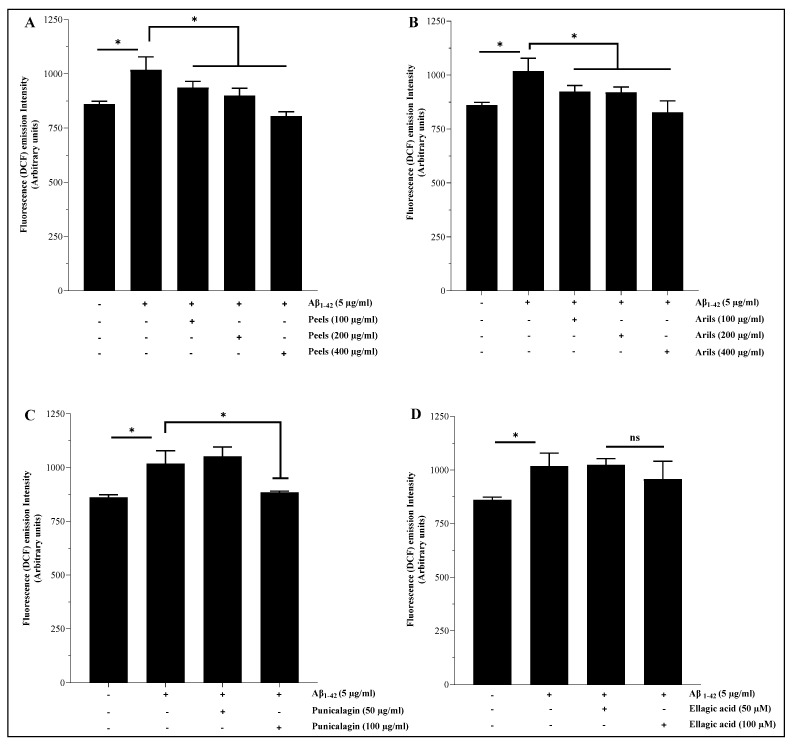
Pomegranate polyphenols reduce ROS generation in human microglia HMC3 cells. Cells were pretreated with human Aβ_1-42_ (5 µg/mL) in the presence, or not, of different concentrations of pomegranate peels (**A**), arils (**B**), punicalagin (**C**) and ellagic acid (**D**). The obtained data are presented as mean ± SEM. (*) vs. Aβ_1-42_: * *p* < 0.05.

Excessive generation of reactive oxygen species (ROS) induces pathological changes in susceptible biomolecules, such as lipids, proteins, and DNA. Oxidative modifications in the structure/function of these vital molecules may cause a series of redox-related diseases, including neurological disorders [73]. Therefore, maintaining the balance between pro/antioxidants is fundamental for cellular homeostasis and disease prevention. We attempted to investigate the ability of the human Aβ_1-42_ to induce oxidative stress in microglial cells. The results showed that peels at 400 µg/mL can significantly (*p* < 0.05) reduce Aβ_1-42_-induced oxidative stress in HMC3 microglia (Figure 4) in a dose-dependent manner (Figure S3A). The peel is well known for its antioxidant properties, and the reported effect may result from synergistic effects between the potent antioxidant polyphenols contained in the peel extract, such as hydrolysable tannins and flavonoids. According to a study published by Subash et al., PPs effectively reduced neuronal oxidative changes in a transgenic mouse model of AD [74]. The authors observed a significant reduction in lipid peroxidation and protein carbonyl, as well as improvements in superoxide dismutase, glutathione peroxidase, and catalase in the treated group. Mechanistically, antioxidant molecules can use their free hydroxyl groups attached to aromatic rings to reduce oxidative species through hydrogen atom transfer reactions. They can also alleviate oxidative damage by acting as chelating agents [75] or as electron-donating compounds [76]. The active phytochemicals of pomegranate, especially the polyphenols extracted from the peels, have the potential for multi-targeted effects, limiting protein, DNA, and lipid damage. The aril treatments showed a significant trend (*p* < 0.05) of attenuating the induced oxidative stress (Figure 4B). This tendency varied significantly (*p* < 0.05) in a concentration-dependent way (Figure S3B). The punicalagin polyphenols were able to reduce ROS generation only at 100 µg/mL (Figure 4C) and did not follow a dose–response model (*p* > 0.05) (Figure S3C). Under the present conditions, ellagic acid polyphenols were not significantly (*p* > 0.05) effective in reducing the oxidative stress induced by Aβ_1-42_.

### 3.5. Lipid Peroxidation Assay

**Figure 5:**
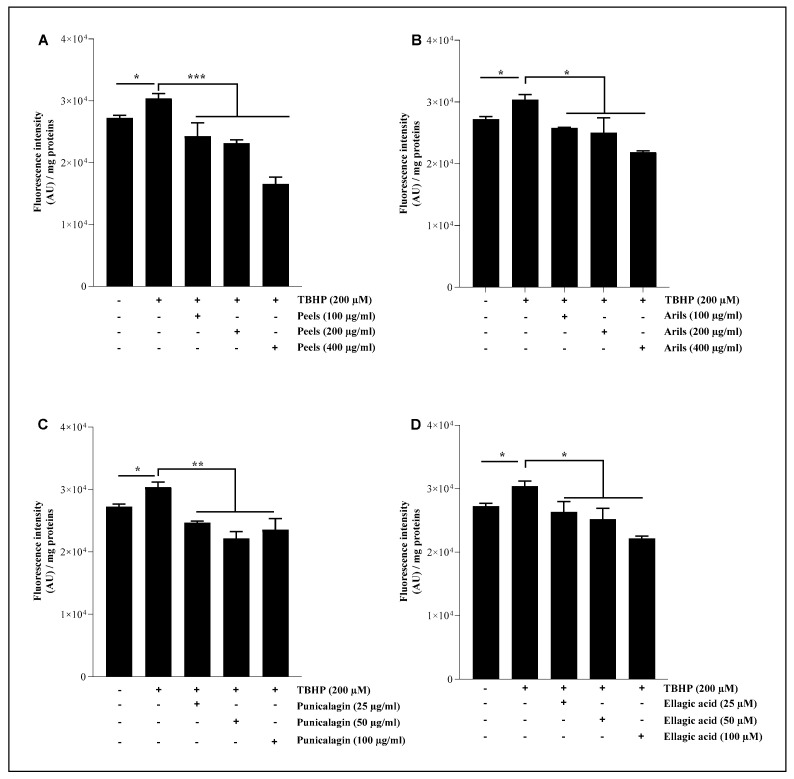
The protective effect of pomegranate polyphenols on lipid peroxidation. HMC3 microglia cells were pretreated (24 h) with peel (**A**) and aril (**B**) extracts, punicalagin (**C**), and ellagic acid (**D**) compounds before their stimulation with TBHP (200 µM) for 1 h. The data are presented as mean ± SEM of at least three independent experiments. * *p* < 0.05; ** *p* < 0.01; *** *p* < 0.001.

Brain lipids contribute to cellular energy capital and essential intermediate effectors in cell signaling pathways [77]. Lipids are also structural components of cell membranes and contribute to cell architecture. They are particular targets of ROS, and the oxidative modifications of lipids that can induce significant dyshomeostasis in the CNS [78]. In this experiment, we pretreated the HMC3 microglial cells with pomegranate treatments for 24 h, followed by TBHP stimulation for 1 h. As illustrated in Figure 5, pomegranate polyphenols were able to significantly (*p* < 0.05) reduce lipid peroxidation in a concentration-dependent manner (Figure S4). The peel polyphenols, at 100, 200, and 400 µg/mL, significantly attenuated (*p* < 0.001) the pro-oxidative effect of TBHP in a dose-dependent manner (Figure S4A). The arils and punicalagin polyphenols showed a similar trend and were significantly (*p* < 0.05 and *p* < 0.01, respectively) effective in decreasing the provoked oxidative stress. The linear regression analysis indicates that this reduction follows significantly (*p* < 0.05) a dose–response trend (Figure S4B,C).

The ellagic acid was only significantly (*p* < 0.05) effective at higher concentrations (100 µM). The peels contain a mixture of powerful antioxidant polyphenolic compounds, such as punicalagin-β and punicalagin-α [75], cyanidin 3-glucoside, pelargonidin 3-glucoside, cyanidin 3,5-diglucoside [79], and quercetin [80], which are known for their antioxidant activities. In vivo evidence, achieved by Morzelle et al. [81], demonstrated that pomegranate peel extract can reduce lipid peroxidation in male C57BI/6 mice. Similarly, Kim et al. [44] reported that 4 weeks of administration of 1.5 mg/kg/day of punicalagin polyphenol to male ICR mice decreased the hydrogen peroxide and MDA levels and increased the GSH/GSSG ratio. These results are comparable to those obtained by Yaidikar et al. [82], who reported that pomegranate downregulates the malondialdehyde level. Furthermore, in vivo evidence obtained by Subash et al. [74] suggests that dietary supplementation with 4% pomegranate results in a significant reduction in lipid peroxidation. The preventive role of pomegranate polyphenols against neurodegeneration could be due to the corrective effect of redox imbalance related to the richness of pomegranate in antioxidant molecules.

### 3.6. M1/M2 Polarization of Microglia and Systemic Macrophages

It has been shown that microglia polarization plays a contributing role in AD progression [83]. In disease conditions, microglia express several proinflammatory cytokines and cell surface inflammatory markers, including CD16, CD32, and CD86, leading to a state of chronic neuroinflammation [84]. To exert their protective effects at the peripheric and central levels, pomegranate polyphenols should reach the bloodstream and cross the BBB. Previous scientific investigations showed the ability of pomegranate polyphenols, especially punicalagin and ellagic acid, to reach human blood. According to Cerdá et al. [85], punicalagin compounds have been detected in the plasma of Sprague–Dawley rats at concentrations (C-max) of around 30 µg/mL. Similarly, ellagic acid was detected at a concentration (C max) of 31.9 ng/mL in human plasma after the consumption of 180 mL of pomegranate juice [86]. Furthermore, Serdar et al. [87] have demonstrated that punicalagin and ellagic acid can cross the BBB in an in vitro model. Similarly, an in vivo evaluation of the BBB penetrability of ellagic acid indicated its capacity to reach the neuronal compartment 0.5–4 h after a dose of 50 mg/kg orally [88]. 

**Figure 6:**
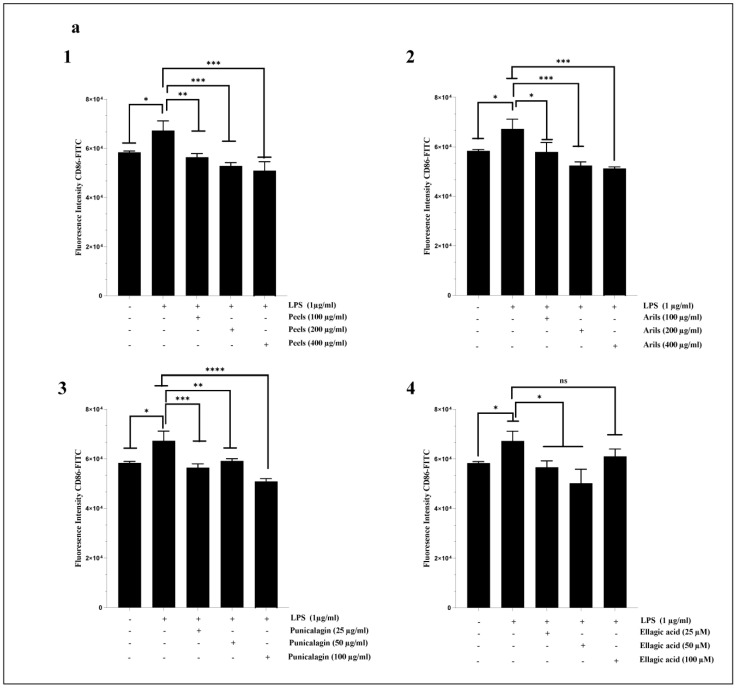

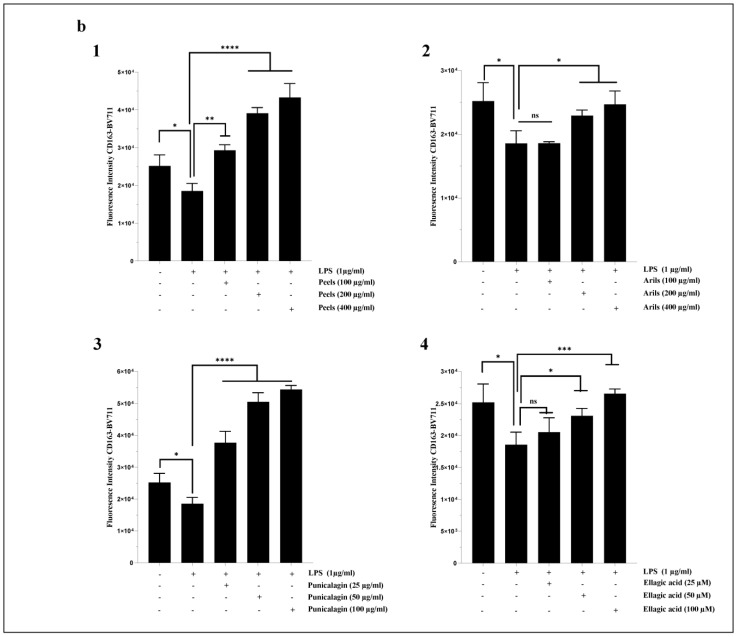
The modulatory effects of peels, arils, punicalagin, and ellagic acid on M1/M2 polarization of HMC3 microglia cells. HMC3 cells were stimulated by 1 µg/mL of LPS and cotreated simultaneously with pomegranate peels, arils, punicalagin, and ellagic acid for 24 h. (**a**) represents the effect of PPs on CD86 expression. (**1**–**4**) illustrates respectively, the effect of pomegranate peels, arils, punicalagin and ellagic acid on CD86 expression. (**b**) illustrates the impact of PPs on CD163 receptor expression. (**1**–**4**) represents the effect of pomegranate peels, arils, punicalagin, and ellagic acid on gene expression of CD163. (*) vs. LPS: * *p*< 0.05; ** *p*< 0.01; *** *p*< 0.001; **** *p*< 0.0001.

Neuroinflammation mediated by the M1 phenotype of microglia is crucial in the neurodegenerative process. Therefore, we attempted to evaluate the ability of our treatments to drive the shift of macrophages from the M1 proinflammatory/destructive phenotype to the M2 anti-inflammatory/protective state. The chronic M1 phenotype is associated with increased release of proinflammatory cytokines and can induce neuronal damage and neurotoxicity. The M2 phenotype is more likely to be associated with increased expression of anti-inflammatory markers and is thought to support neuronal survival and cellular homeostasis. We report that LPS significantly (*p* < 0.05) upregulates CD86 expression in HMC3 microglia cells (Figure 6a). This finding agrees with results obtained by Lu et al. [89], who reported an increase of around 29% in CD86 expression in HMC3. In our experiment, the pomegranate treatments showed a trend of mitigating M1-related neurotoxicity by decreasing the expression of the cell surface receptor CD86 and increasing CD163 protein expression (Figure 6). The peel-rich phenolic extract dose-dependently reduced the CD86 expression (*p* < 0.001) (Figure 6(a1) and Figure S5(1A)). The peel treatment was also dose-dependently (*p* < 0.0001, Figure S5(2A)) effective in improving the M2 phenotype through CD163 upregulation (Figure 6(b1)). It has been demonstrated that pomegranate peel polyphenols target the suppression of the TLR4/NF-κB pathway to inhibit LPS-induced inflammation [90]. Peel extract contains a mixture of polyphenolic constituents recognized for driving anti-inflammatory pharmacological activities, such as ellagitannin bioactive compounds [91]. We have demonstrated that aril phenolic extract also modulated LPS-induced neuroinflammation (Figure 6 and Figure S5). However, arils appear less potent than peels in modulating the M1/M2 phenotypic transition in microglia. The previous publication by our team [42] could explain this difference between peel and aril extracts. Our team previously demonstrated that peel extracts are richer in antioxidant and anti-inflammatory compounds, such as punicalagin polyphenols, than aril extracts [42].

Similarly, the incubation of cells with punicalagin resulted in a significant (*p* < 0.0001) shift of microglial HMC3 cells from the M1 to the M2 phenotype via the downregulation of CD86 (Figure 6(a3)) and upregulation of CD163 (Figure 6(b3)) cell surface receptor expression. The linear regression analysis suggests that the mentioned effect of punicalagin polyphenols follows a concentration-dependent model (Figure S5). Ellagic acid is a natural antioxidant polyphenol and a powerful punicalagin metabolite that gives rise to anti-inflammatory urolithin constituents [92], and it was reported to drive various neuroprotective activities [93]. In our study, we demonstrated that ellagic acid could modulate the proinflammatory state of microglia by decreasing (in a non-dose–response manner (*p* > 0.05); Figure S5(1D)) the expression of CD 86 (Figure 6(a4)) and increasing in a concentration-dependent manner (Figure S5(2D)) that of CD163 (Figure 6(b4)). 

Only one previously published study [33] attributed comparable effects to cyanidin-3-O-glucoside, an anthocyanin compound found in many vegetables and fruits, including pomegranate. According to the authors, cyanidin-3-O-glucoside could shift the M1 phenotype of HMC3 microglial cells to the M2-protective phenotype, as it can decrease the expression of CD86 and CD80, inflammatory cytokines (IL-1β, IL-6, and TNF-α), and oxidative stress, as well as increase M2-specific markers, particularly CD206 and CD163. These results were confirmed by the same authors under in vivo conditions, using APPswe/PS1ΔE9 mice. However, there are no previous reports on the modulatory effect of punicalagin, ellagic acid, pomegranate peels, and aril polyphenols on microglia (M1/M2) polarization. 

**Figure 7:**
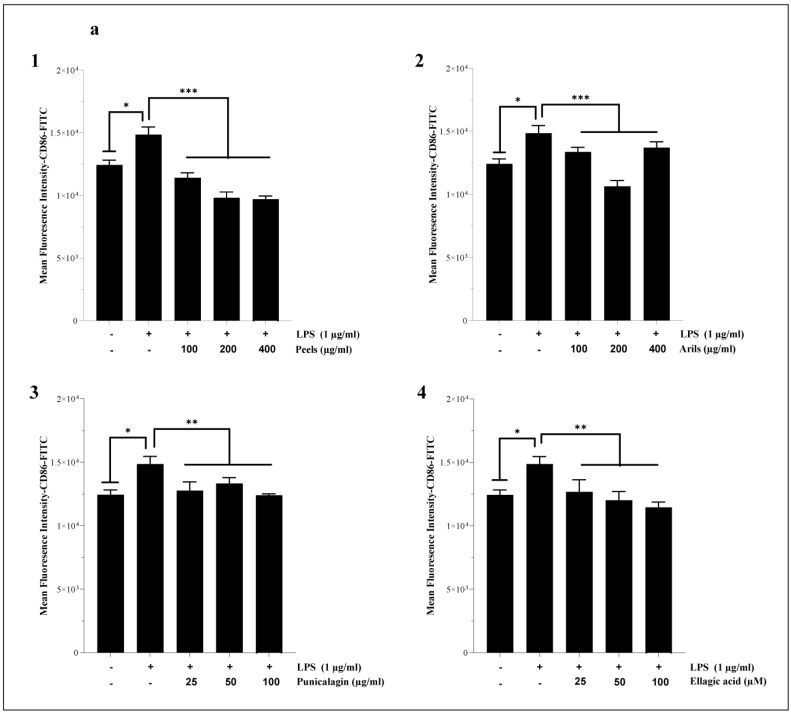

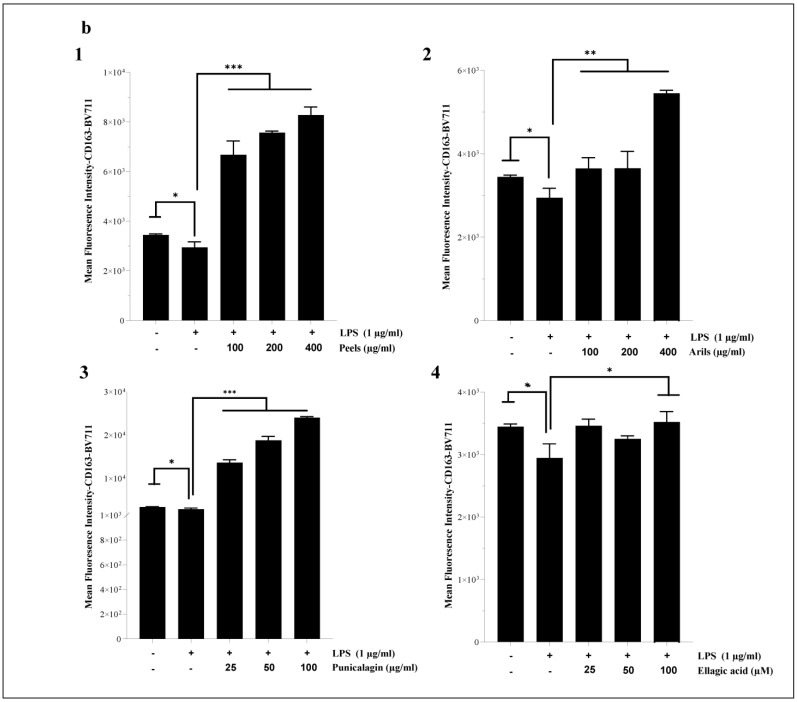

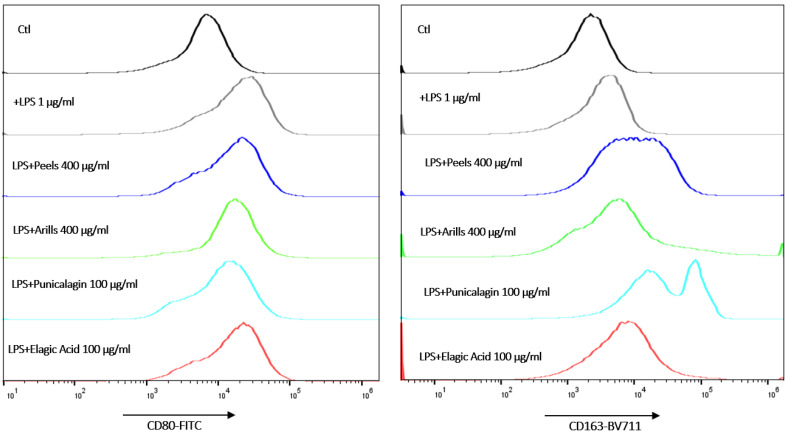
The effect of pomegranate polyphenols on CD86 (**a**) and CD163 (**b**) protein expression. Human-THP-1-derived macrophages were stimulated by LPS (1 µg/mL) and cotreated with pomegranate peels (**1**), aril-rich phenolic extracts (**2**), punicalagin (**3**), and ellagic acid (**4**), for 24 h. (*) vs. LPS: * *p*< 0.05; ** *p*< 0.01; *** *p*< 0.001.

Systemic chronic inflammation is involved in the etiology of many incurable diseases [94,95], including atherosclerosis and AD. In the present paper, we first investigated the response of human systemic THP-1 macrophages to LPS stimulation. Secondly, we attempted to modulate the resulting M1 phenotype by using increasing concentrations of the used PPs. Our data suggest that LPS significantly (*p* < 0.05) enhances the expression of CD86 in THP-1 macrophages and, therefore, corroborate those previously published by Hennen et al. [96]. The results shown in Figure 7(a1) indicate that the peels significantly (*p* < 0.001) and dose-dependently (*p* < 0.0001, Figure S6(1A)) downregulated CD86 receptor expression. This is in agreement with the obtained data from Xin-Yu Lu and coworkers [97], who reported that pomegranate peel extracts can reduce inflammation through the reduction in the percentages and absolute numbers of CD80^+^ and CD86^+^ cells. Pomegranate peels are rich in hydrolysable tannins and various gallic acid esters, which are widely recognized as mediating anti-inflammatory activities [98,99,100,101]. The aril-rich phenolic extract was also significantly effective in reducing CD86 expression, notably at a concentration of 200 µg/mL (*p* < 0.001, Figure 7(a2)). This effect follows a dose–response relationship (*p* < 0.0001, Figure S6(1B)). Arils contain various active substances, including flavonoids and anthocyanins [102]. These active phytochemicals are endowed with anti-inflammatory properties [103,104]. In addition, punicalagin significantly (*p* < 0.001) reversed the M2-to-M1 phenotypic transition of THP-1 macrophages induced by LPS stimulation. The punicalagin reduced CD86 expression (Figure 7(a3)) in a dose-dependent manner (*p* < 0.01, Figure S6(1C)). It was reported that punicalagin treatments can promote an M2-like macrophage polarization via the upregulation of HO-1 in murine macrophages [105], and could be a preventive strategy against inflammatory disorders. Furthermore, ellagic acid significantly (*p* < 0.01) attenuated the LPS-induced M1 phenotype in THP1 macrophages and downregulated, in a concentration-dependent manner (*p* < 0.01, Figure S6(1D)), CD86 protein expression (Figure 7(a4)). Recent in vivo research [106] has revealed similar findings, suggesting that ellagic acid at 50 and 100 µM attenuated LPS-induced neuroinflammation. These effects may be exerted directly, by ellagic acid, or indirectly, by its metabolites, including urolithin A, which can easily cross into the neuronal microenvironment [107]. 

The effect of our treatments on CD163 expression is illustrated in Figure 7b. The reported findings indicate a powerful modulation of the THP-1 macrophage state. The incubation of cells with LPS resulted in a significant (*p* < 0.05) decrease in CD163 cell surface receptors (Figure 7b). This downregulation was corrected by the peel polyphenols, which significantly (*p* < 0.001) increased the CD163 receptors (Figure 7(b1)) in a concentration-dependent manner (Figure S6(2A); *p* < 0.0001). The aril treatments, especially at 400 µg/mL, remarkably and positively shifted the expression of CD163 in a concentration-dependent way (*p* < 0.01) (Figure 7(b2) and Figure S6(2B)). A similar trend was observed with the punicalagin polyphenols, which upregulated, in a dose-dependent manner (Figure S6(2C); *p* < 0.0001), CD163 protein expression, and the most powerful effect was achieved at 100 µg/mL (Figure 7(b3)). On the other hand, it appears that the ellagic acid at 25 and 50 µM was not able to significantly improve CD163 M2 markers (*p* > 0.05), while at a higher concentration, its effect was significant (100 µM; *p* < 0.05) (Figure 7(b4)). Comparable findings were also obtained by Aharoni et al. [108], using a J774-A1 macrophage-like cell line. The authors of that study reported that punicalagin and pomegranate juice polyphenols could promote macrophages’ switch to an anti-inflammatory M2 response [108]. Furthermore, pomegranate juice can limit age-associated switching from M2 to M1 in mice [108]. 

Together, the reported findings and the discussed literature point to a significant protective transition from M1 to M2 polarization mediated by PPs both at the central and the peripheric levels. These add additional support to the central role of polyphenols from natural resources in contracting significant inflammatory alterations that occur in atherosclerosis and AD.

### 3.7. The Assessment of Interleukin 1-Beta (IL-1β) and Interleukin-10 (IL-10) Cytokines Expression

Inflammation is a crucial mechanism of innate immunity that aims to guide, enhance, and accelerate the healing process. However, chronic inflammation is harmful to neurons, and it can lead to irreversible degeneration, causing a progressive decline in vital brain functions. Various scans of the AD brain showed a high level of inflammatory markers, and numerous genome-wide association studies suggested that several immune-related loci can increase susceptibility to AD [109]. On the other hand, many nonsteroidal anti-inflammatory agents (NSAIDs) can reduce the risk of developing AD [110,111]. This evidence, among other findings, points to the potential involvement of the immune system in the etiology of AD. This paper evaluates the possible inflammation-lowering effects of pomegranate peels, arils, punicalagin, and ellagic acid in U373-MG human astrocytes and THP1 monocytes differentiated into systemic macrophages. We quantified the expression of proinflammatory cytokines (Il-1β) and anti-inflammatory markers (IL-10) by flow cytometry. 

**Figure 8:**
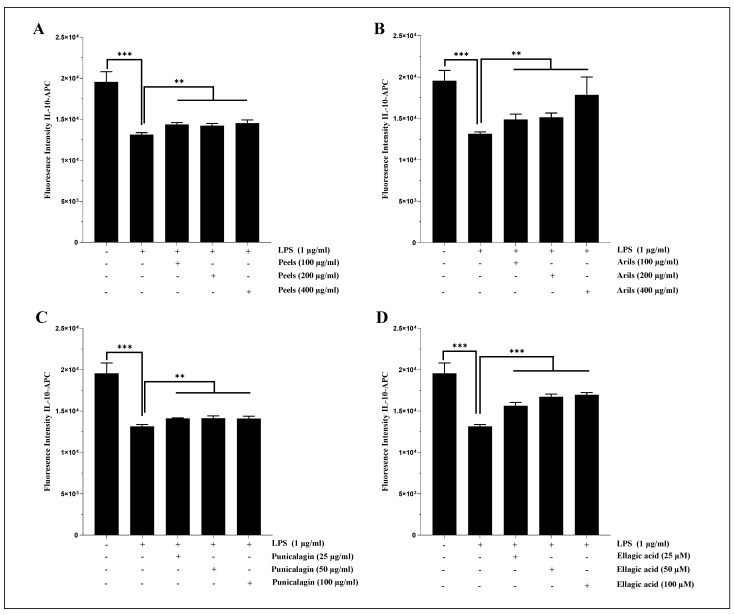
The bioeffects of pomegranate polyphenols on gene expression of IL-10. The U373-MG human astrocytes were stimulated by 1 µg/mL of LPS and cotreated simultaneously overnight with pomegranate peels (**A**), arils, (**B**) punicalagin (**C**), and ellagic acid (**D**) at different concentrations. (*) vs. LPS: ** *p*< 0.01; *** *p*< 0.001.

In the U373-MG astrocytes, the LPS (1 µg/mL) significantly (*p* < 0.001) reduced the expression of IL-10 (Figure 8). These proinflammatory effects were attenuated in a dose-dependent manner by the peel (100–200 and 400 µg/mL, *p* < 0.01; Figure 8A and Figure S7A) and aril treatments (Figure 8B and Figure S7B). Similarly, the punicalagin (Figure 8C) significantly (25–50 and 100 µg/mL, *p* < 0.01) reduced, in a dose-dependent manner (Figure S7C; *p* < 0.05), the cytotoxicity of LPS-induced neuroinflammation and significantly increased the expression of IL-10 cytokines in the human astrocyte cells. Furthermore, the ellagic acid treatment upregulated (Figure 8D), dose-dependently (Figure S7D; *p* < 0.0001), IL-10 expression. Previous in vivo studies reported PPs as potent glial inflammatory process regulators. Findings obtained by [81] and by [47] suggested that peel-rich phenolic extract can reduce TNF-α and Il-1β expression both in C57BI/6 and in APPsw/Tg2576 mice. Similarly, punicalagin suppresses Il-1β, IL-6, and TNF-α in male ICR mice [44]. Moreover, a short-term intervention (14 days) using urolithin A, an ellagic acid derivative, significantly downregulated the gene expression of IL-1β, IL-6, and TNF-α in a transgenic female model that expressed APP/PS1 mutation [112]. From a mechanistic point of view, PPs can act through IKK and Ikb inhibition [44,46], decrease NF-kB DNA binding activity, and reduce p50 and p65 subunit translocation [44,46].

**Figure 9:**
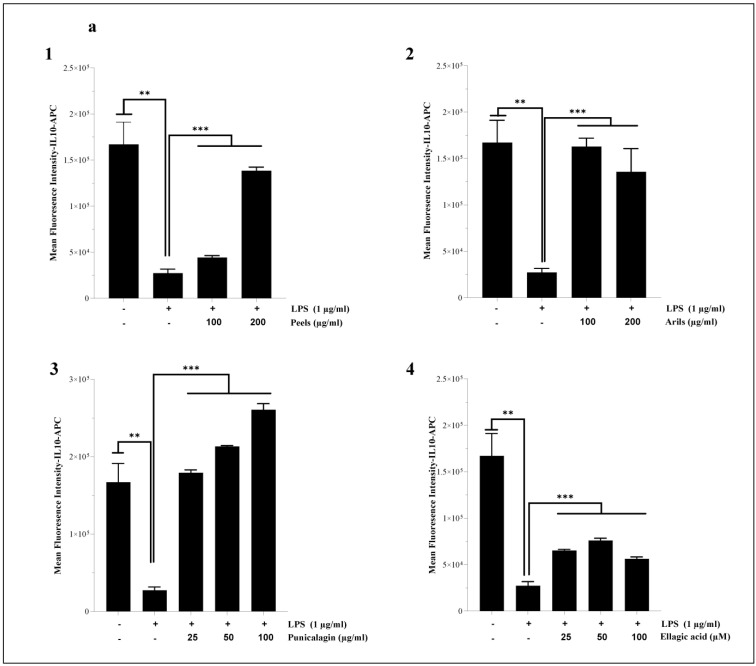

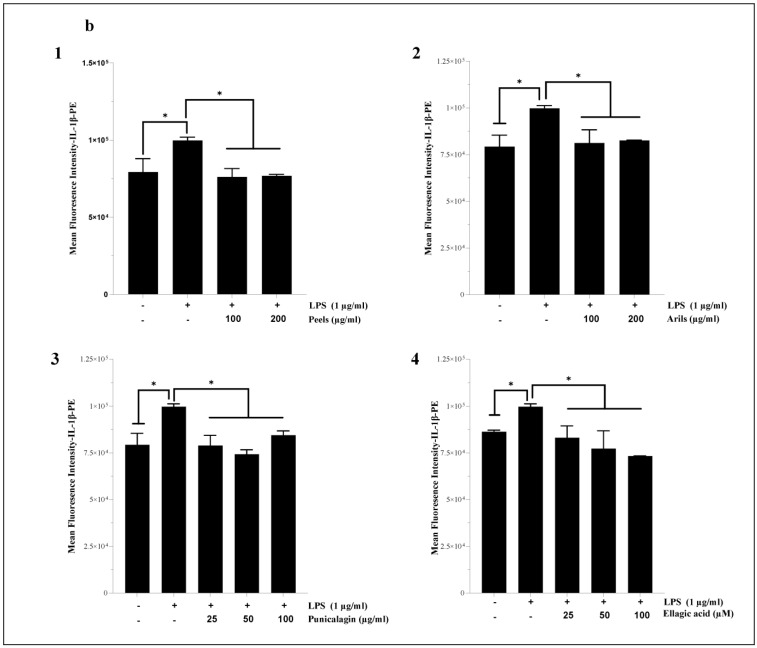
The modulatory effects of pomegranate peels (**1**), arils (**2**), punicalagin (**3**), and ellagic acid (**4**) on IL-10 (**a**) and IL-1β (**b**) protein expression in THP-1-derived macrophages. Cells were stimulated by 1 µg/mL of LPS and co-treated simultaneously with pomegranate polyphenols for 24 h. (*) vs. LPS: * *p* < 0.05; ** *p* < 0.01; *** *p* < 0.001.

We also found that pomegranate treatments can alleviate LPS-induced systemic inflammation in human THP1 monocytes differentiated into macrophages. Indeed, LPS stimulation (1 µg/mL) resulted in a significant (*p* < 0.001) decrease in IL-10 cytokine expression (Figure 9a). The induced inflammation was significantly (*p* < 0.001) ameliorated by the peels and arils (Figure 9(a1) and Figure 9(a2), respectively), as well as being drastically suppressed (*p* < 0.001) in a concentration-dependent manner by the punicalagin (*p* < 0.0001) (Figure 9(a3) and Figure S8(1C)) and ellagic acid (*p* < 0.05) (Figure 9(a4) and Figure S8(1D)). In the same sense, the incubation of THP-1 macrophages with LPS resulted in a significant (*p* < 0.05) increase in IL-1β proinflammatory cytokines in comparison to the untreated group (Figure 9b). The polyphenols from pomegranate approximatively suppressed the proinflammatory effects of LPS. The peels, arils, and punicalagin approximatively returned (*p* < 0.05, Figure 9(b1), Figure 9(b2), and Figure 9(b3), respectively), in a dose-dependent manner (*p* < 0.05), THP-1 macrophages to the basal state (Figures S8(2A), S8(2B), and S8(2C), respectively), while the ellagic acid reduced in a very remarkable way the expression of IL-1β, especially at 100 µM (Figure 9(b4)). 

Our data join the previously published scientific reports [90,113] on the anti-inflammatory effects mediated by pomegranate polyphenols. Indeed, it has been reported that punicalagin from a pomegranate can inhibit (NF-κB and MAPK) activation in response to LPS stimulation in RAW264.7 macrophages, and enhances LC3II and p62 protein expression [113]. Furthermore, a study published by Du et al. [90] found peel extract’s suppressive activities against the TLR4/NF-κB pathway. The preventive and corrective effects of pomegranate against other neurodegenerative diseases are debated, as Tapias et al. [114] reported essential and unexpected results in a rotenone model of Parkinson’s disease. The authors showed adverse effects of PPs, such as dopaminergic neuronal death, aggravation of the inflammatory response, and caspase activity enhancement. This may have been due to the high concentration used, as polyphenols are recognized as promoting proinflammatory pathways at high doses [115]. However, the reported unexpected findings were not confirmed in a recent study published by Kujawska et al. [116], who suggested opposite findings. To our knowledge, no published data exist regarding the effect of ellagic acid and arils on the investigated cytokines. Additionally, human findings related to the modulatory effects of PPs on neuroinflammation are not available. This shows a deep gap and the considerable need to examine the reported in vitro and the discussed in vivo beneficial effects on human beings.

In general, PPs appear to be effective in mitigating Aβ_1-42_-induced neuroinflammation. This may explain why the use of anti-inflammatory drugs, particularly NSAIDs, has been associated in previous studies with a lower risk of developing dementia [21,22]. These anti-inflammatory agents likely act in a similar way by reducing Aβ_1-42_-related toxicity. Consequently, it will be interesting to evaluate the effect of combining these drugs with PPs, not only for a possible enhancement of their efficacy, but also for a potential reduction in their side effects.

### 3.8. Tau Phosphorylation at Threonine 181

**Figure 10:**
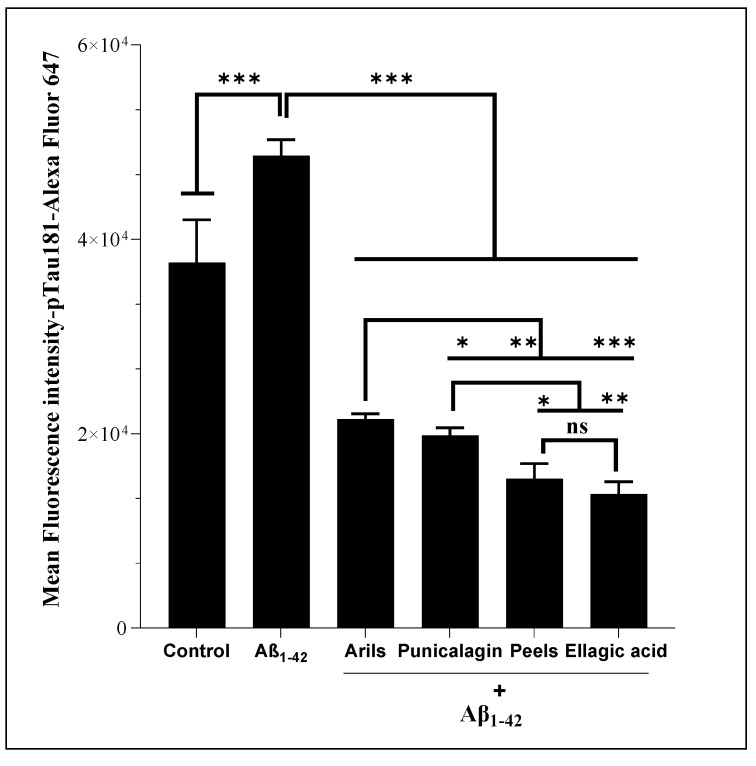
The modulatory effects of pomegranate polyphenols on human Aβ_1-42_-induced Tau phosphorylation at threonine 181 in H4 human neuroglioma cells. Cells were either unstimulated or stimulated with human Aβ_1-42_ (10 µg/mL) in the presence or absence of pomegranate polyphenols simultaneously for 24 h. Peels: 200 µg/mL; arils: 200 µg/mL; punicalagin: 50 µg/mL; ellagic acid: 50 µM. (*) vs. Aβ_1-42_: * *p* < 0.05; ** *p* < 0.01; *** *p* < 0.001.

Various scientific investigations have associated the abnormal phosphorylation of Tau protein with AD development [117,118,119]. From a mechanistic point of view, Tau phosphorylation can be involved in neurodegeneration via the alteration of Tau-related physiological functions, such as microtubule-binding activities [120] and neurite outgrowth [121,122]. In addition, much clinical evidence has been found for elevated hyperphosphorylated Tau in the brains of patients suffering from AD [123], and this was correlated with cognitive decline in several published papers [124,125]. In our study, we performed the flow cytometry analysis, first, to examine whether human Aβ_1-42_ can induce the phosphorylation of Tau protein at threonine 181 and, second, to evaluate the modulatory impact of our treatments against Aβ_1-42_-induced Tau phosphorylation. We report that incubating H4 neurons with human Aβ_1-42_ resulted in a significant (*p* < 0.001) increase in pTau-181. A previous analysis reported that Aβ_1-42_ stimulation significantly increased the phosphorylation of Tau (by 1.8-fold) [126]. Our intervention using PPs significantly suppressed (*p* < 0.0001) the Aβ_1-42_-stimulating effect on Tau phosphorylation (Figure 10). The pomegranate peels and ellagic acid appear to be more potent than the other treatments (*p* < 0.05), followed by the punicalagin and aril (*p* < 0.05) polyphenols. It has been shown that ellagic acid can ameliorate learning and memory impairments by inhibiting Aβ and Tau phosphorylation [127]. Our findings could explain this effect, and we suspect the indirect impact of ellagic acid on p-Tau via the reduction of Aβ-production. Furthermore, possible direct interactions between PPs, particularly ellagic acid and Tau kinases/phosphatases, including glycogen-synthase kinase-3β (GSK3β), cyclin-dependent kinase 5 (CdK5), and cAMP-dependent protein kinase (PKA) [128,129], could explain this dynamic regulation of Tau. 

Other polyphenols, including quercetin-3-O-glucuronide, gallic acid, and isoorientin, demonstrated potent neuroprotection against Aβ_1-42_ neurotoxicity [130,131,132]. Recent findings from Xu and colleagues [130] suggest that quercetin-3-O-glucuronide can alleviate cognitive deficit by reducing Tau phosphorylation, neuroinflammation, and cell apoptosis in Aβ_1-42_-induced AD-like mice. Similarly, Ding et al. [131] suggest that gallic acid may promote neurogenesis and reduce cognitive impairment in an APP/PS1 mouse model. The authors linked this improvement to gallic acid’s ability to enhance synaptic plasticity and decrease Aβ_1-42_ and Tau phosphorylation burden. Furthermore, isoorientin, a natural C-glycosyl flavonoid, has been shown to reverse synaptic dysfunction, reduce Aβ_1-42_ levels/Aβ_1-42_-deposition, decrease Tau protein hyperphosphorylation, and reduce microglia activation in the brains of APP/PS1 mice [132].

In terms of the effects of total phenolic extracts, numerous research groups have demonstrated, on a clinical scale, the usefulness of these polyphenols in mediating neuroprotective activities. In this sense, a double-blind, randomized, placebo-controlled trial published by Akhondzadeh et al. [133] suggests that melissa officinalis-rich phenolic extract could attenuate episodic memory disorders and improve cognitive capacity in patients suffering from mild to moderate AD. Simularly, Choudhary and coworkers [134] reported that an eight-week intervention with *Withania somnifera* (L.) *Dunal* root extract resulted in a significant improvement in memory and cognitive functions. This neuroprotection might also be secondary to improving other physiologic functions via mutual interactions between these polyphenols, gut microbiota, mineral metabolism, and neuronal cells [130,135,136]. 

Natural substances and diets rich in polyphenols can drive positive impacts and limit AD-associated Tau pathology. 

It has been shown that polyphenols can interact synergistically to potentiate their health benefits. Indeed, Mitra and colleagues reviewed the experimental proof of this ability in a recent paper [137]. The authors of this article were able to identify fifteen molecules that can act synergistically. These include gallic acid, kaempferol, quercetin, and cyanidin. These polyphenols naturally occur in pomegranates [138,139,140,141], which can contribute to explaining why, in some conditions, the used phenolic extracts, especially the peel extract, produced a more significant effect than the pure molecules.

## 4. Conclusions and Perspectives

The current study provides direct evidence to support the beneficial effects of PPs in Tau pathology against oxidative stress and neuroinflammation-mediated neurodegeneration. However, several limitations should be highlighted and taken into account in future studies:

First, the molecular mechanisms and signaling pathways solicited by PPs to mitigate Aβ_1-42_ neurotoxicity have not been determined. To address this particular point, further investigations should focus on examining the possible role of PPs in Tau kinases (CdK5 and GSK-3β) and targeting the NF-κB- and NLRP3-inflammasome pathways. 

Secondly, the translational gaps between the present in vitro findings and clinical applications must be addressed by using AD-relevant models and conducting well-designed randomized placebo-controlled trials.

Thirdly, the discussed neuroprotective effect of PPs is critically dependent on their ability to reach the neuronal compartment. Consequently, the interaction between these polyphenols and the BBB could influence their effectiveness. Therefore, we emphasize the need for more studies to assess the BBB penetrability of the used molecules.

Future work should also determine the most effective, safest dose and dose–response relationships, as well as the potency of the pomegranate bioactive compounds. In this context, we suggest, as a perspective, investigating the gut (microbiome)-based polyphenol metabolites of ellagic acid, especially the urolithin constituents, as they showed higher BBB penetrability and a diverse array of neuroprotective actions with multi-targeted physiological effects. 

## Data Availability

Data are contained within the article.

## Supplementary Materials

The following supporting information can be downloaded at: https://www.mdpi.com/article/10.3390/nu16213667/s1, Figure S1. The effect of human Aβ1-42 on H4 neurons viability. H4 cells were seeded in 96 well-plates for 24 h to stabilize, and then were stimulated or not with increasing concentrations (5 to 20 µg/ml) of human Aβ1-42 for an additional 24h. Results are expressed as mean ± SEM. **** *p* < 0.0001, indicates the level of significance in comparison with the control group; Figure S2. The effect of 24 h of incubation with pomegranate peels and arils on H4 cell viability. Cells were incubated with or without increasing concentrations of pomegranate peels (100; 200; and 400 µg/mL) or pomegranate arils (100; 200; and 400 µg/mL); Figure S3. The linear regression of the Figure 4, related to the effect of pomegranate polyphenols on ROS generation in human microglia HMC3 cells; Figure S4. Linear regression of Figure 5, related to the protective effect of pomegranate polyphenols on lipid peroxidation; Figure S5. The linear regression of Figure 6, related to the effect of peels, arils, punicalagin, and ellagic acid on M1/M2 polarization of HMC3 microglia cells; Figure S6 Linear regression of Figure 7, related to the effect of the effect of pomegranate polyphenols on CD86 (Figure 7a) and CD163 (Figure 7b) in THP-1-derived macrophages; Figure S7. Linear regression of the Figure 8, related to the bioeffects of pomegranate polyphenols on gene expression of IL-10 in U373-MG human astrocytes; Figure S8. Linear regression of Figure 9, related to the the modulatory effects of pomegranate polyphenols on on IL-10 (Figure 9a) and IL-1β (Figure 9b) protein expression, in THP-1-derived macrophages.
