# Supplementary material for: In Vitro Assessment of the Neuroprotective Effects of Pomegranate (Punica granatum L.) Polyphenols Against Tau Phosphorylation, Neuroinflammation, and Oxidative Stress
Source: Nutrients. 2024 Oct 28;16(21):3667. doi: 10.3390/nu16213667 (PMC11547808; doi:10.3390/nu16213667)
Supplement: Supplementary file 1 [file nutrients-16-03667-s001.zip › nutrients-3233687-supplementary.pdf]

Supplementary Data

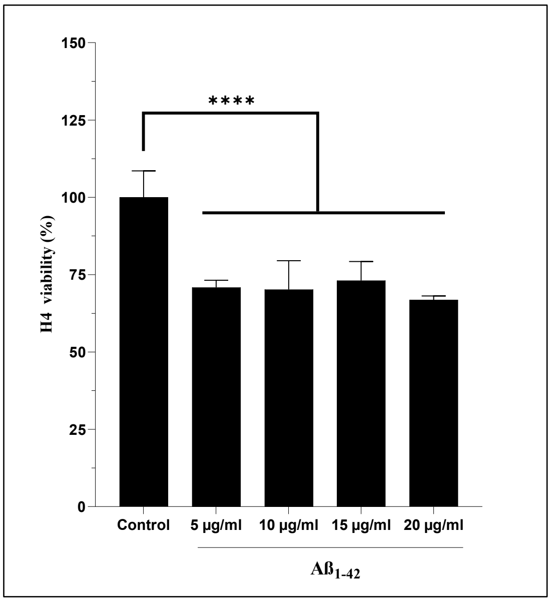

Figure S1. The effect of human Aβ_{1-42} on H4 neurons viability. H4 cells were seeded in 96 well-plates for 24 h to stabilize, and then were stimulated or not with increasing concentrations (5 to 20 µg/ml) of human Aβ_{1-42} for an additional 24h. Results are expressed as mean ± SEM. ****$p < 0.0001$, indicates the level of significance in comparison with the control group.

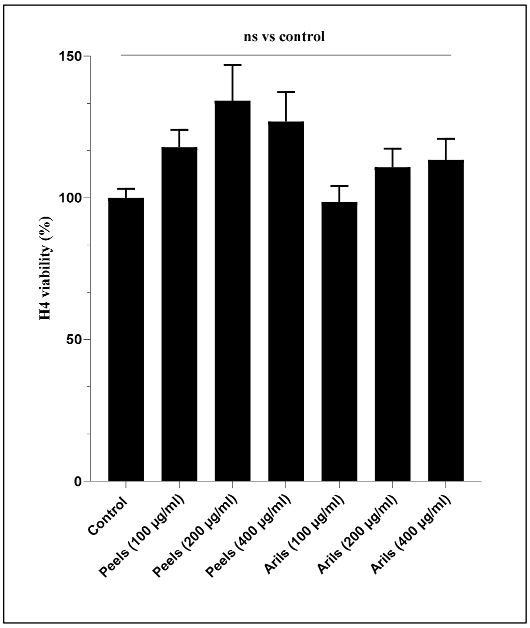

Figure S2. The effect of 24 h of incubation with pomegranate peels and arils on H4 cell viability. Cells were incubated with or not with increasing concentrations of pomegranate peels (100; 200; and 400 µg/ml) or pomegranate arils (100; 200; and 400 µg/ml).

Linear Regression

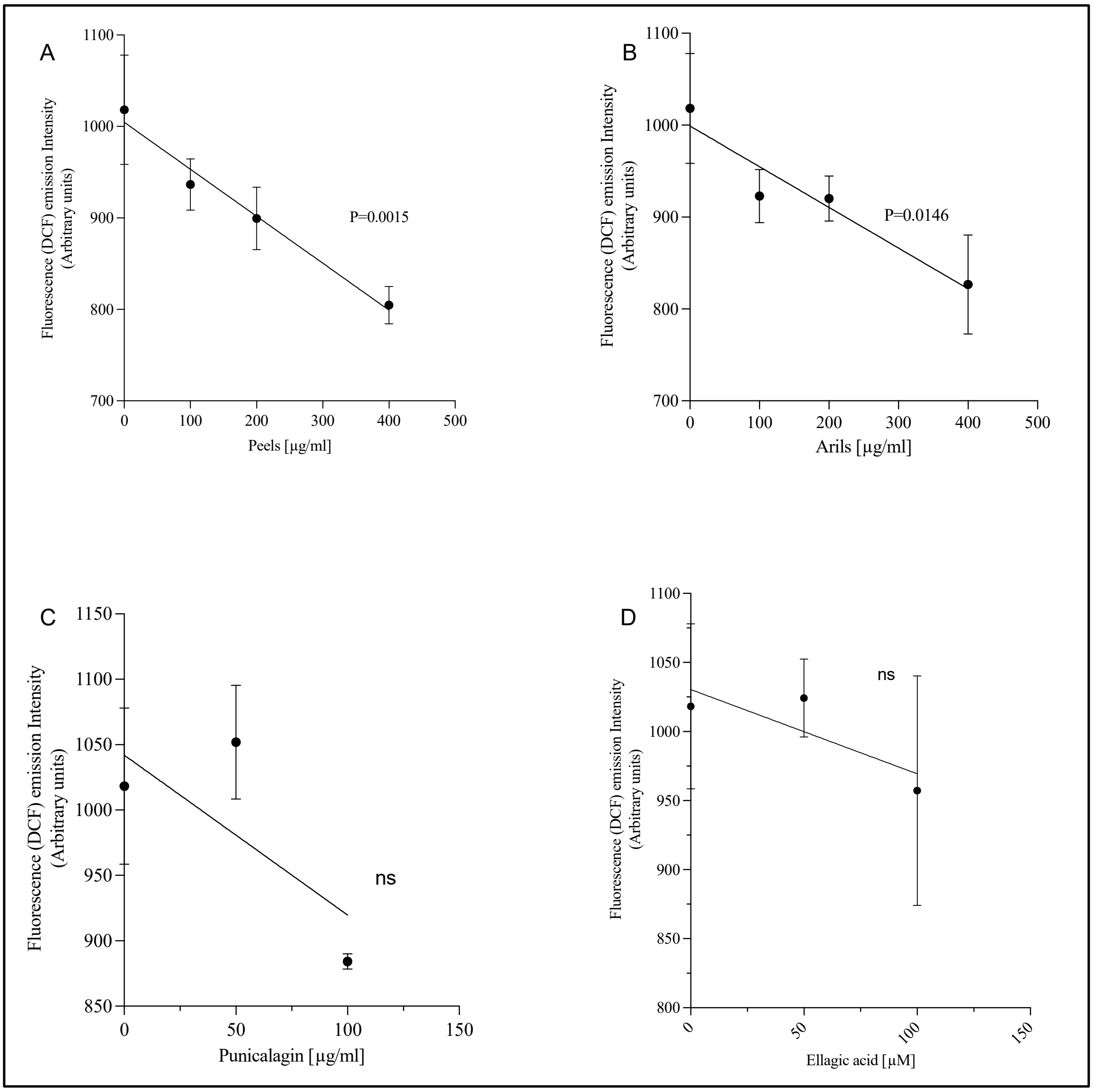

Figure S3. The linear regression of the **Figure 4**, related to the effect of pomegranate polyphenols on ROS generation in human microglia HMC3 cells.

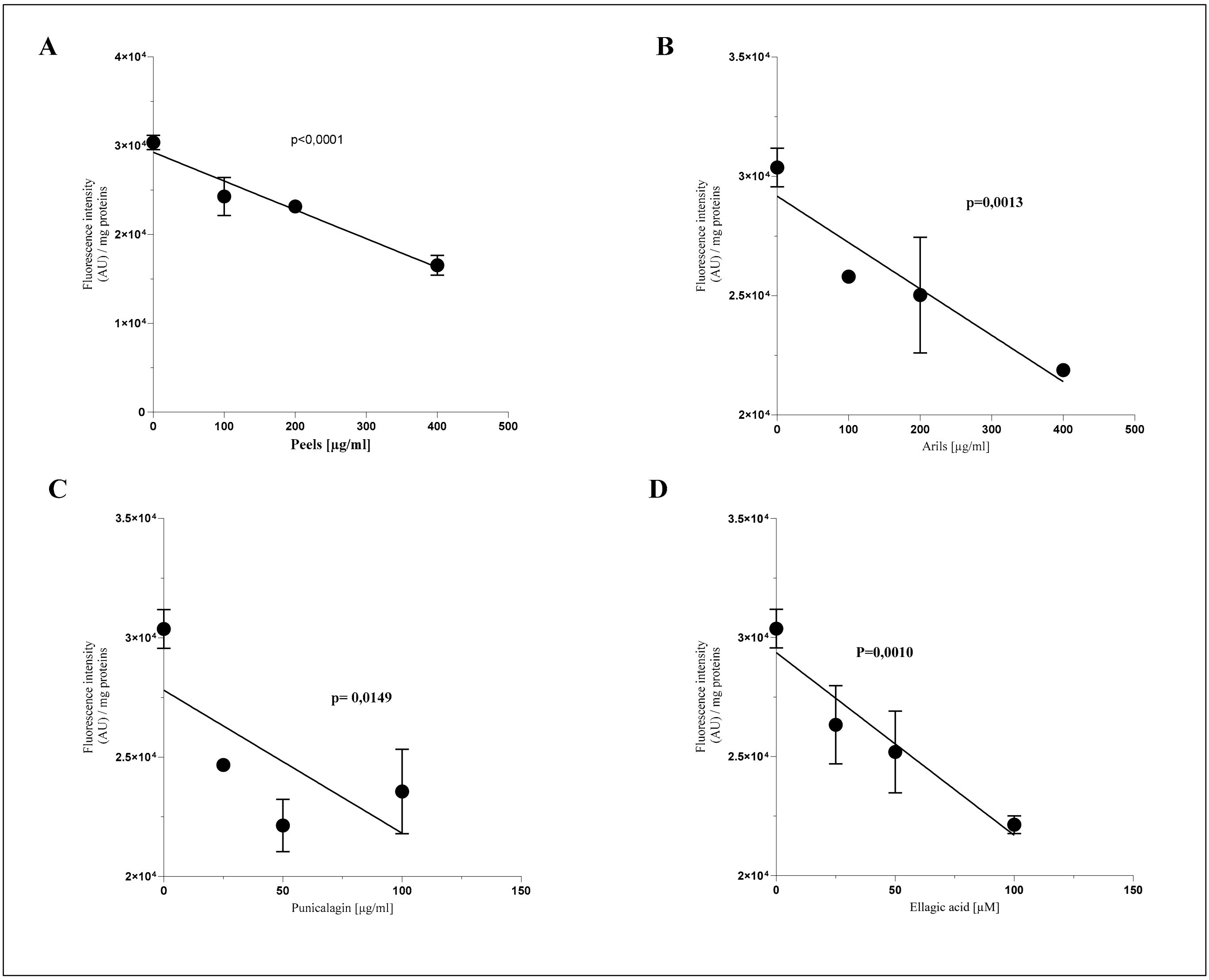

Figure S4. linear regression of **Figure 5**, related to the protective effect of pomegranate polyphenols on lipid peroxidation.

Figure S5.1:

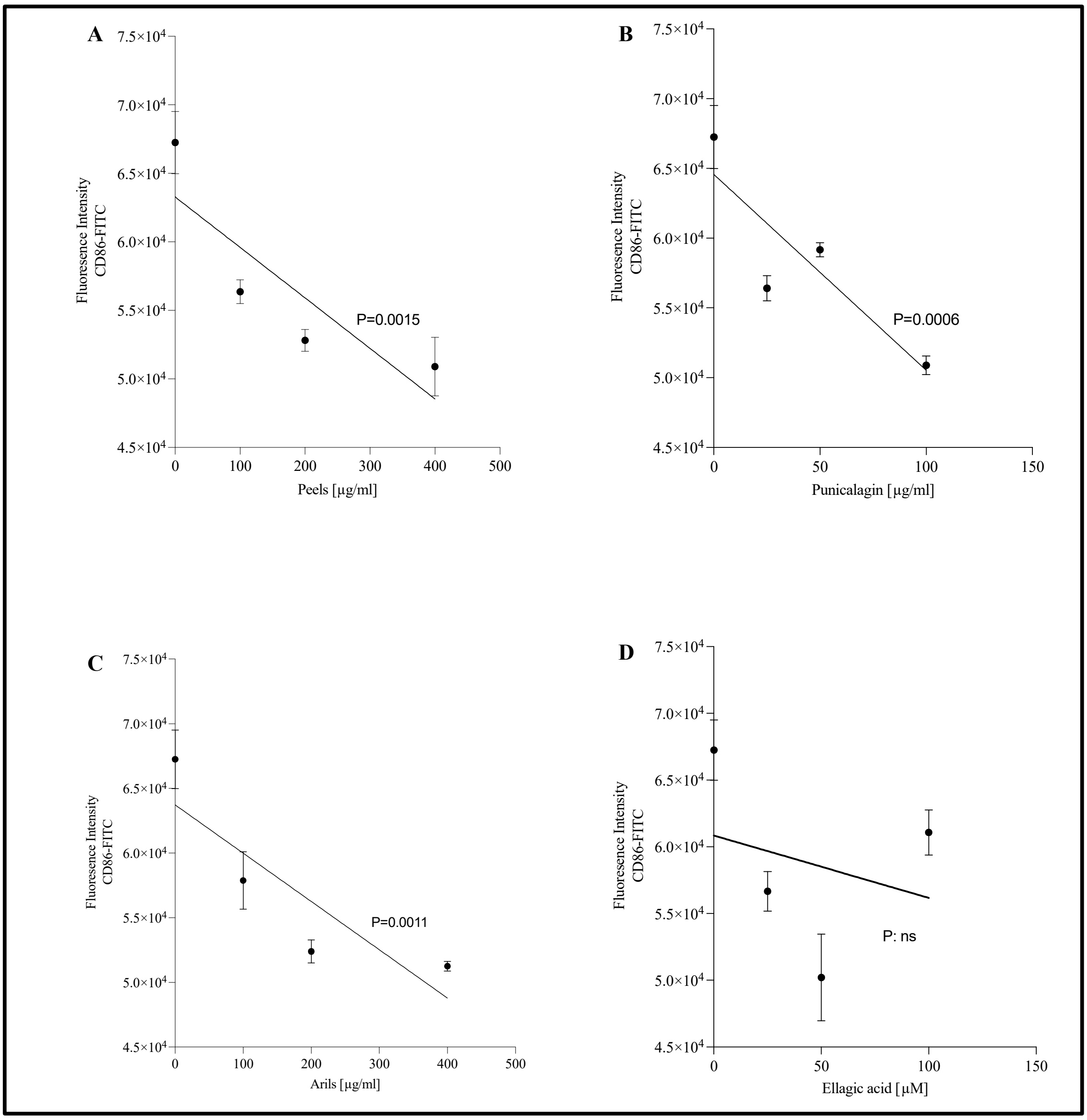
A
7.5×10⁴
7.0×10⁴
6.5×10⁴
6.0×10⁴
5.5×10⁴
5.0×10⁴
4.5×10⁴
Fluoresence Intensity
CD86-FITC
P=0.0015
0
100
200
300
400
500
Peels [µg/ml]
B
P=0.0006
0
50
100
150
Punicalagin [µg/ml]
C
P=0.0011
Arils [µg/ml]
D
P: ns
Ellagic acid [µM]

Figure S5.2:

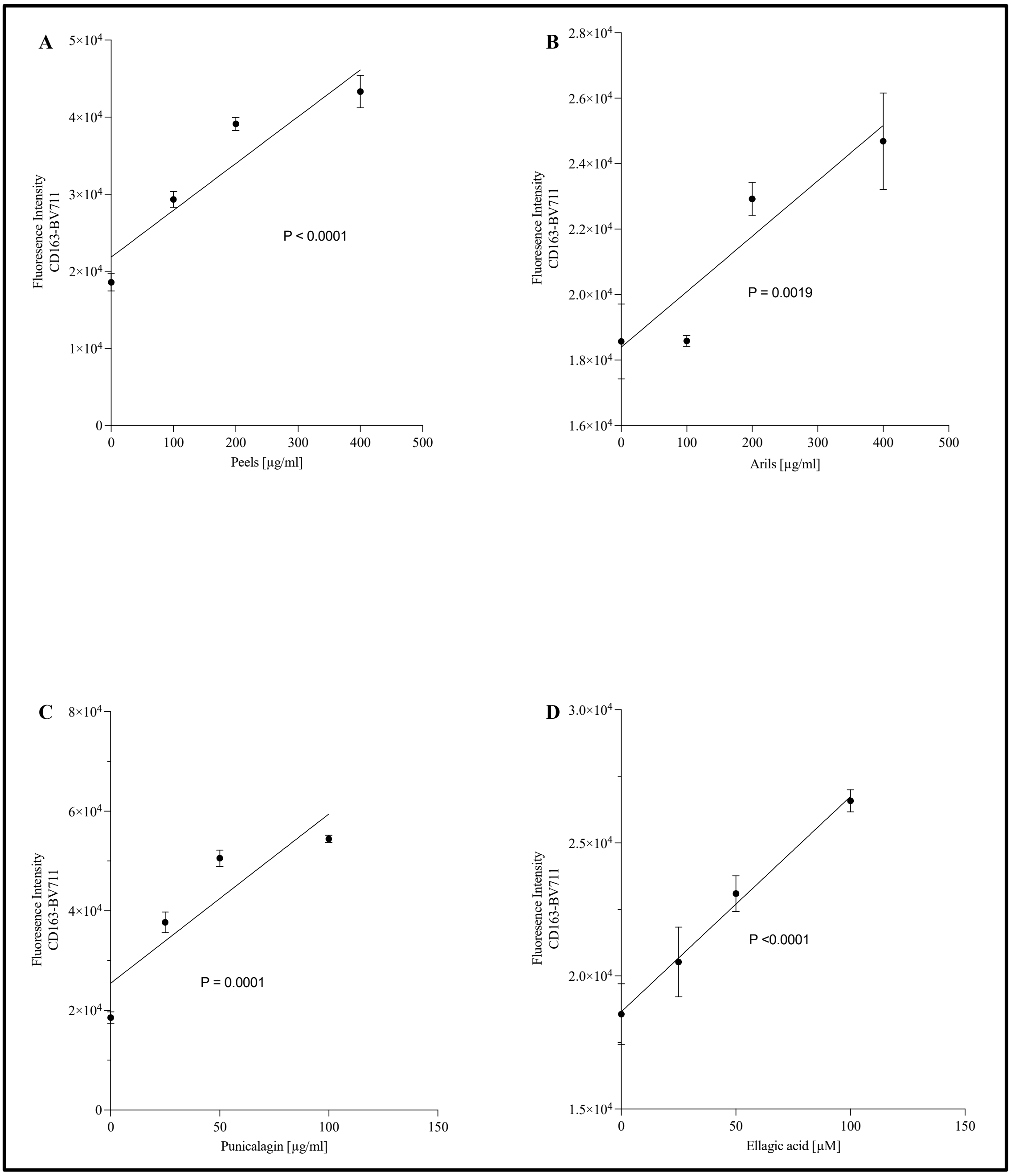

Figure S5. the linear regression of **Figure 6**, related to the effect of peels, arils, punicalagin, and ellagic acid on M1/M2 polarization of HMC3 microglia cells. **Figure S5.1:** represents the linear regression of **Figure 6a**; and the **Figure S5.2:** represents the linear regression of **Figure 6b**.

Figure S6.1:

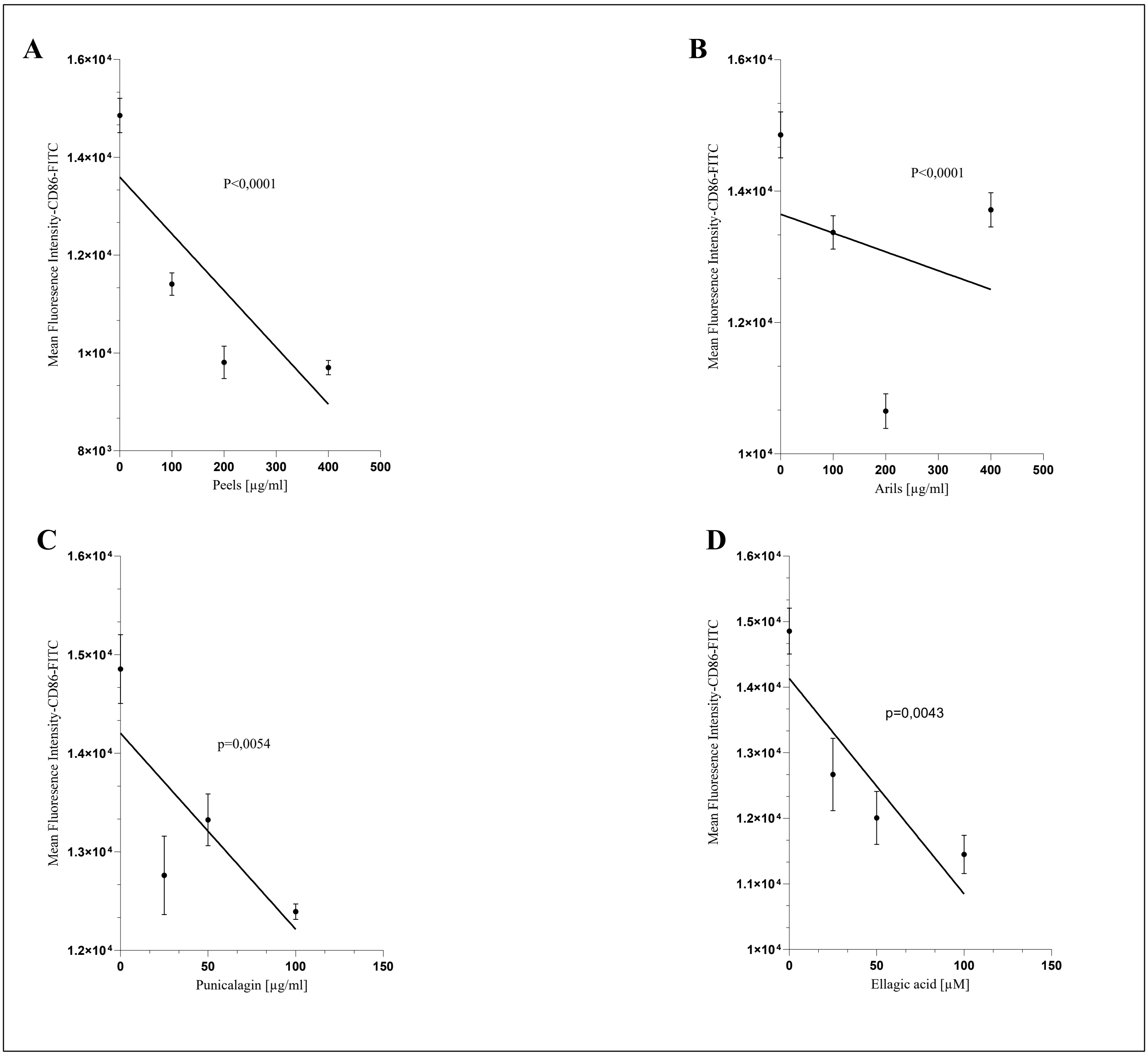
A
1.6×10⁴
1.4×10⁴
1.2×10⁴
1×10⁴
8×10³
0
100
200
300
400
500
Mean Fluoresence Intensity-CD86-FITC
Peels [µg/ml]
P<0,0001
B
1.6×10⁴
1.4×10⁴
1.2×10⁴
1×10⁴
Mean Fluoresence Intensity-CD86-FITC
Arils [µg/ml]
P<0,0001
C
1.6×10⁴
1.5×10⁴
1.4×10⁴
1.3×10⁴
1.2×10⁴
0
50
100
150
Mean Fluoresence Intensity-CD86-FITC
Punicalagin [µg/ml]
p=0,0054
D
1.6×10⁴
1.5×10⁴
1.4×10⁴
1.3×10⁴
1.2×10⁴
1.1×10⁴
1×10⁴
Mean Fluoresence Intensity-CD86-FITC
Ellagic acid [µM]
p=0,0043

Figure S6.2:

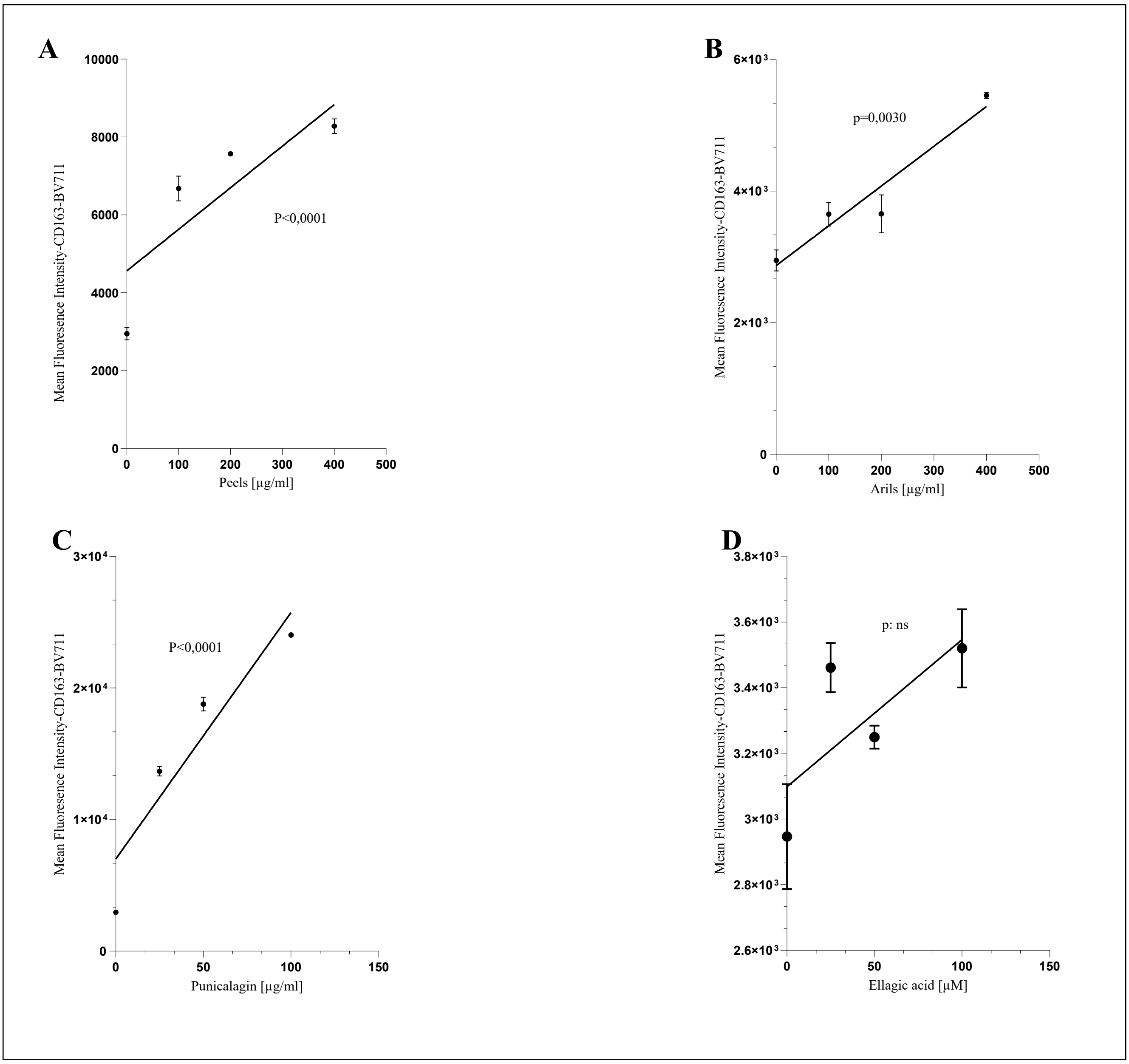

Figure S6. Linear regression of **Figure 7**, related to the effect of the effect of pomegranate polyphenols on CD86 (**Figure 7a)** and CD163 (**Figure 7b)** in THP-1-derived macrophages. **Figure S6.1:** represents the linear regression of **Figure 7a**; and the **Figure S6.2**, illustrates the linear regression of **Figure 7b**.

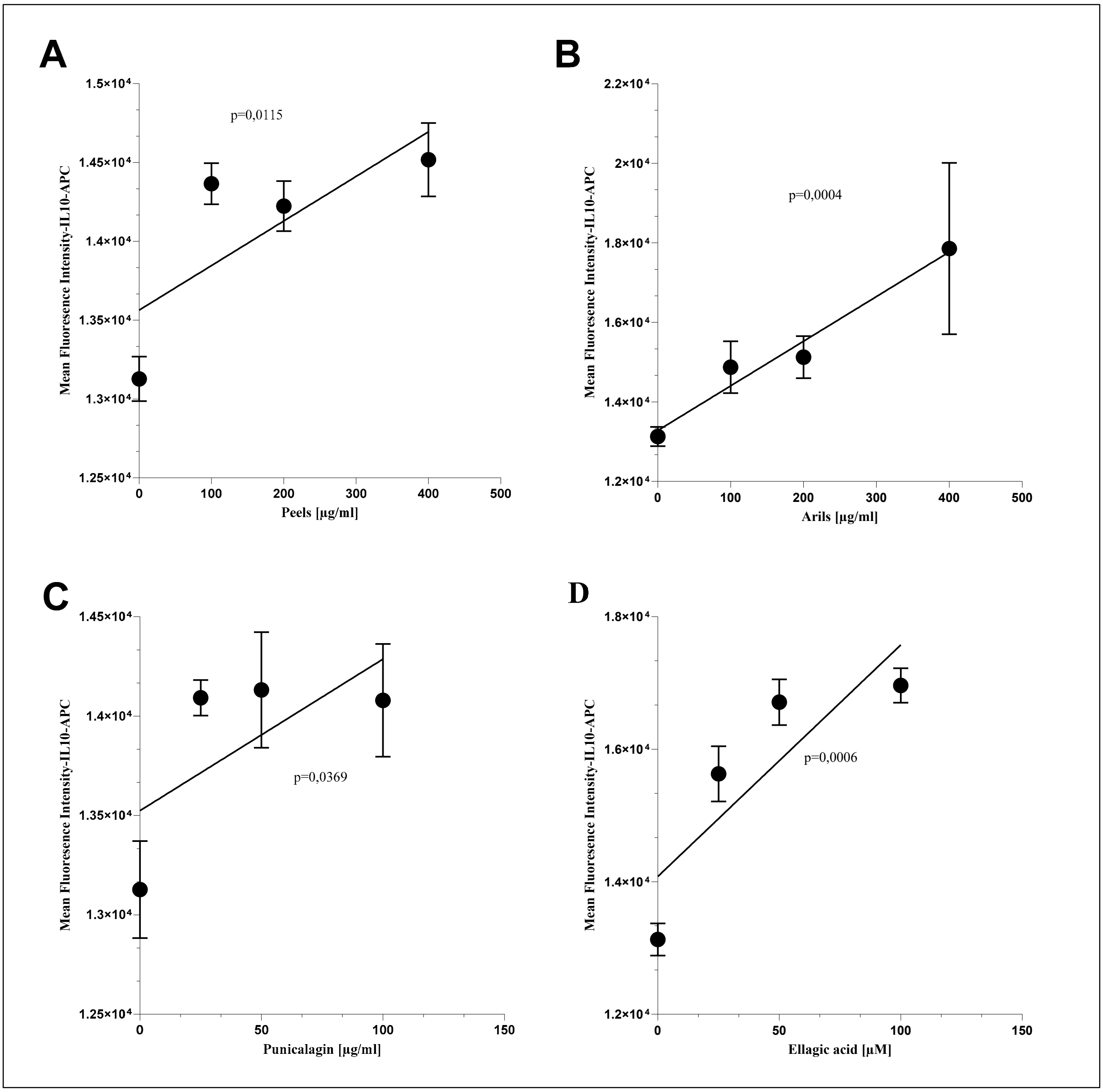

Figure S7. linear regression of the **Figure 8**, related to the bioeffects of pomegranate polyphenols on gene expression of IL-10 in U373-MG human astrocytes.

Figure S8.1:

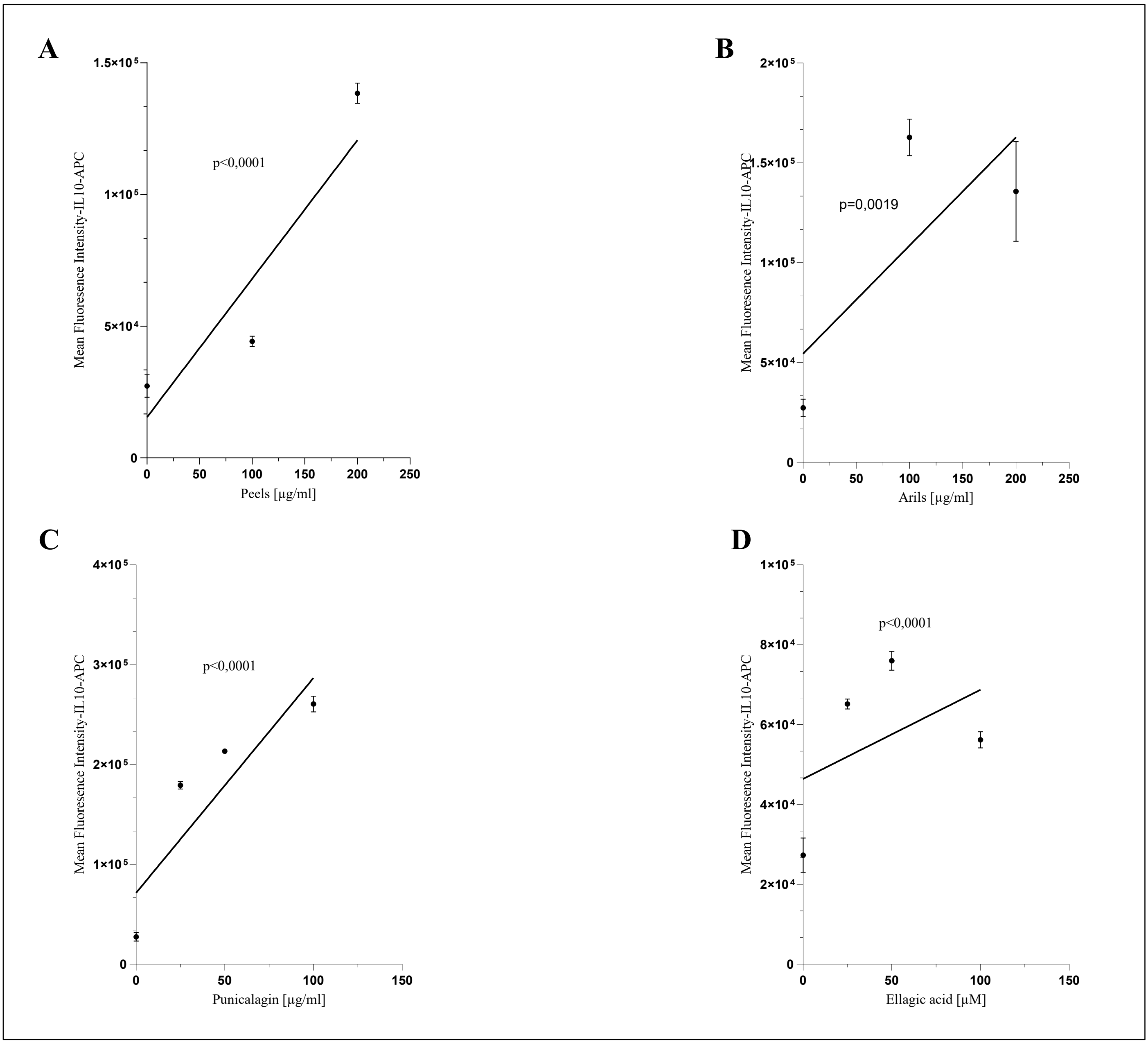
A
1.5×10⁵
1×10⁵
5×10⁴
0
p<0,0001
Mean Fluoresence Intensity-IL10-APC
0 50 100 150 200 250
Peels [µg/ml]
B
2×10⁵
1.5×10⁵
1×10⁵
5×10⁴
0
p=0,0019
Mean Fluoresence Intensity-IL10-APC
0 50 100 150 200 250
Arils [µg/ml]
C
4×10⁵
3×10⁵
2×10⁵
1×10⁵
0
p<0,0001
Mean Fluoresence Intensity-IL10-APC
0 50 100 150
Punicalagin [µg/ml]
D
1×10⁵
8×10⁴
6×10⁴
4×10⁴
2×10⁴
0
p<0,0001
Mean Fluoresence Intensity-IL10-APC
0 50 100 150
Ellagic acid [µM]

Figure S8.2:

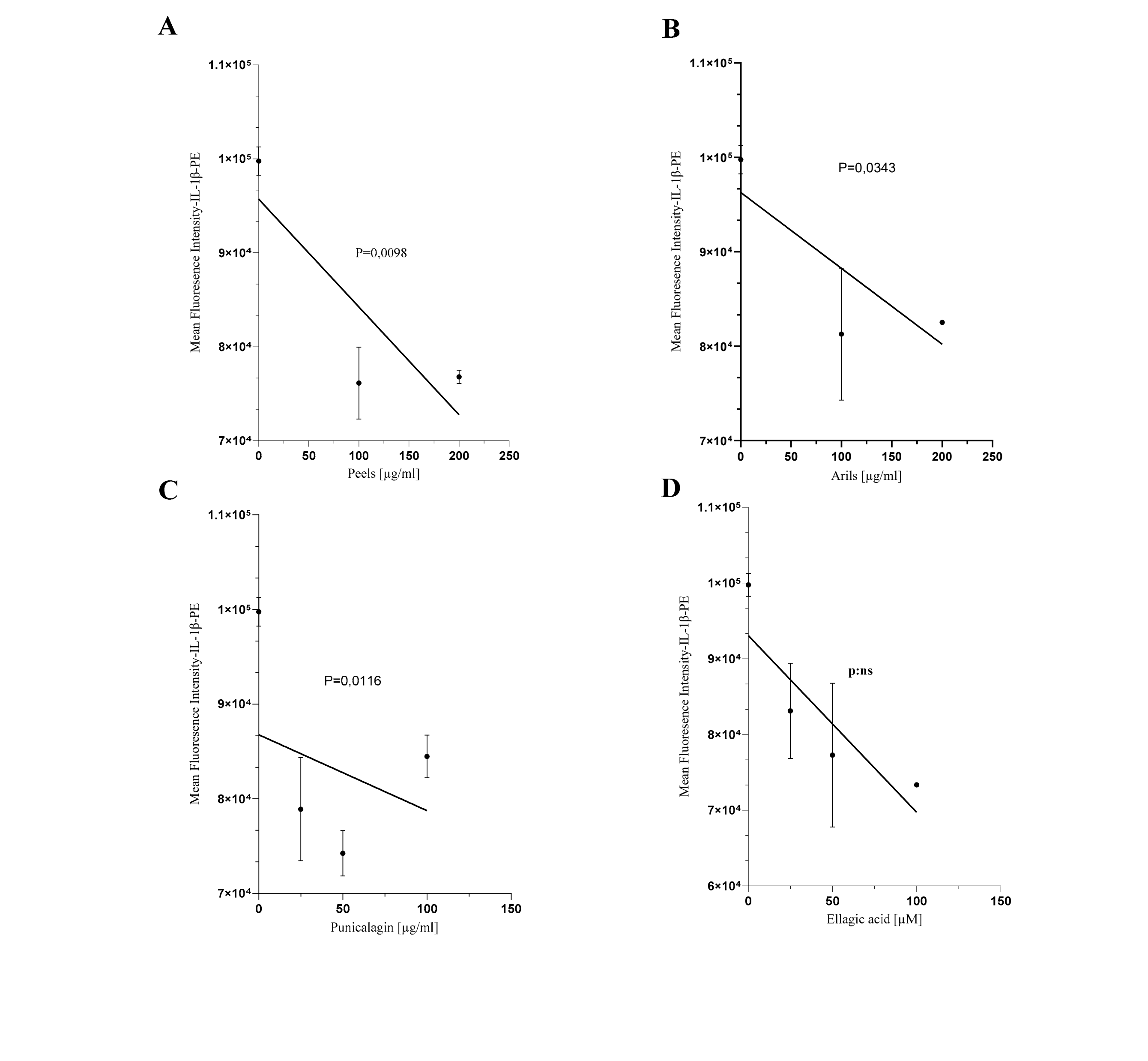

Figure S8. Linear regression of **Figure 9,** related to the the modulatory effects of pomegranate polyphenols on on IL-10 (**Figure 9.a**) and IL-1β (**Figure 9.b**) protein expression, in THP-1-derived macrophages.. **Figure S8.1**: represents the linear regression of **Figure 9a**; and the **Figure S8.2**: illustrates the linear regression of **Figure 9b**.
